# Dynamic microbiome diversity shaping the adaptation of sponge holobionts in coastal waters

**DOI:** 10.1128/spectrum.01448-24

**Published:** 2024-10-14

**Authors:** Bifu Gan, Kai Wang, Beibei Zhang, Chenzheng Jia, Xin Lin, Jing Zhao, Shaoxiong Ding

**Affiliations:** 1 State Key Laboratory of Marine Environmental Science, College of Ocean and Earth Sciences, Xiamen University, Xiamen, China; 2 Xiamen City Key Laboratory of Urban Sea Ecological Conservation and Restoration (USER), Xiamen University, Xiamen, China; Oulun yliopisto, Oulu, Finland

**Keywords:** sponge holobiont, symbiotic microbial community, coastal zones, temporal variation, environmental adaptative strategy, meta-analysis of microbiome

## Abstract

The microbial communities associated with sponges contribute to the adaptation of hosts to environments, which are essential for the trophic transformation of benthic-marine coupling. However, little is known about the symbiotic microbial community interactions and adaptative strategies of high- and low-microbial abundance (HMA and LMA) sponges, which represent two typical ecological phenotypes. Here, we compared the 1-year dynamic patterns of microbiomes with the HMA sponge *Spongia officinalis* and two LMA sponge species *Tedania* sp. and *Haliclona simulans* widespread on the coast of China. Symbiotic bacterial communities with the characteristic HMA–LMA dichotomy presented higher diversity and stability in *S. officinalis* than in *Tedania* sp. and *H. simulans*, while archaeal communities showed consistent diversity across all sponges throughout the year. Dissolved oxygen, dissolved inorganic phosphorus, dissolved organic phosphorus, and especially temperature were the major factors affecting the seasonal changes in sponge microbial communities. *S. officinalis*-associated microbiome had higher diversity, stronger stability, and closer interaction, which adopted a relatively isolated strategy to cope with environmental changes, while *Tedania* sp. and *H. simulans* were more susceptible and shared more bacterial Amplicon Sequence Variants (ASVs) with surrounding waters, with an open way facing the uncertainty of the environment. Meta-analysis of the microbiome in composition, diversity, and ecological function from 13 marine sponges further supported that bacterial communities associated with HMA and LMA sponges have evolved two distinct environmental adaptation strategies. We propose that the different adaptive ways of sponges responding to the environment may be responsible for their successful evolution and their competence in global ocean change.

**IMPORTANCE:**

During long-term evolution, sponge holobionts, among the oldest symbiotic relationships between microbes and metazoans, developed two distinct phenotypes with high- and low-microbial abundance (HMA and LMA). Despite sporadic studies indicating that the characteristic microbial assemblages present in HMA and LMA sponges, the adaptation strategies of symbionts responding to environments are still unclear. This deficiency limits our understanding of the selection of symbionts and the ecological functions during the evolutionary history and the adaptative assessment of HMA and LMA sponges in variable environments. Here, we explored symbiotic communities with two distinct phenotypes in a 1-year dynamic environment and combined with the meta-analysis of 13 sponges. The different strategies of symbionts in adapting to the environment were basically drawn: microbes with LMA were more acclimated to environmental changes, forming relatively loose-connected communities, while HMA developed relatively tight-connected and more similar communities beyond the divergence of species and geographical location.

## INTRODUCTION

Microbial symbionts are essential for the health, survival, and function of multicellular eukaryotes, ranging from animals to plants (1). Microbes can adapt quickly to new environments owing to their plastic responses and dynamic nature that provide further pathways for facilitating their association with hosts and tackling environmental threats (2), while hosts, that typically have longer reproductive cycles and smaller population sizes, may be less responsive to environmental changes than microbiomes (3). Diverse symbionts are the result of the long-term evolution of microbes and hosts (4), and the process of interaction between them continuously drives the holobiont to adapt to the surrounding environment. Resilience is thought to be tightly linked to community diversity, and a feature is that the relative abundance of microbial composition has changed but retention of functionality (5). In the broadest sense, resilience describes the ability of an ecosystem to resist and recover from a disturbance (6). We have difficulties in predicting whether environmental changes affect species loss, but we could simply access the biological potential of holobiont to the environment by the resilience and ecological processes of the associated microbial communities. So far, however, the adaptation strategies and environmental drivers of the microbiota remain largely unknown.

Sponge (Phylum Porifera) is considered a typical model of host-microbe interactions in early differentiated metazoan clades dated from nearly 890 million years (7). Sponges harbor diverse and complex microbial communities (8). Moreover, they do not have tissue and organ differentiation and only rely on cell layers to isolate symbiotic microbial assemblages from the aqueous environment (9). The related functional microbiota may play the role of various organs and tissues similar to higher animals, facilitating the host to build adaptive capacity to environmental change. Sponges are common members of marine benthic communities around the world (10). Sponge-associated symbiotic microbial communities have long been highly temporal and spatial stable, and substantially affect the biogeochemical cycling of key nutrients such as carbon, nitrogen, and phosphorous (11–13). In addition, secondary metabolites produced by the sponge microbiome contribute to the immune functions of holobionts in the defense against pathogens, as well as other functions of coral reef ecosystems (6). With coral decline and bleaching due to global warming (14), sponges play an increasingly important role in coral reefs and there is increasing interest in their trophic ecology (15).

The sponge-associated microbes include diverse phylogenetic lineages of bacteria and archaea, fungi, and viruses (16). Sponges have evolved two distinct symbiotic community states—high-microbial abundance (HMA) and low-microbial abundance (LMA) (17). HMA sponges harbor 2–4 orders of magnitude greater densities of microbes than LMA sponges (18). The composition of microbial symbionts reflects the distinctions between HMA and LMA sponges as well (18). A general consensus exists that HMA sponges have a greater microbial diversity than LMA (18, 19). At the phylum level, the HMA microbiome is more diverse, with Proteobacteria, Chloroflexi, Acidobacteria, Actinobacteria, and other phyla acting as major community members (18, 19). However, the LMA microbiome is dominated by Proteobacteria (α-, β-, or γ-) or Cyanobacteria (genus *Synechococcus*), with little overlap among the investigated microbiome (18, 19). The distinction between LMA and HMA sponges reflects two different life-history strategies. LMA sponges showed a higher pumping rate, which rapidly took up large volumes of seawater through their tissues, and mainly absorbed and transformed particulate organic matter (POM), while HMA sponges showed a denser mesohyl and a lower pumping rate, and relied more on dissolved organic matter (DOM) (20, 21). Host-associated microbiomes are shaped by various interacting selective forces in the holobiont (4); however, the mechanisms of microbial selection and the resulting strategies of adaptation to the environment in HMA and LMA sponges are currently unclear.

Symbiosis is context dependent, and the context is rapidly changing. Whether HMA or LMA sponges are important members of marine ecosystems, the stability and resilience of these ecosystems are thought to be strongly dependent upon symbiotic communities (8). Sponge holobionts are gradually viewed as models for studying complex combinations of stable and transient assemblies in marine environments (22). The adaptability of sponges to the environment is closely related to many thousands of stable core microbes (4). However, dramatic environmental changes may lead to symbiosis breakdown and even partner death (23). Changes in environmental temperature (24) or heavy metal stress (25) can modify the microbial community structure, weaken the interaction between sponges and microorganisms, and reduce nutrient exchange and defense capacity, which poses significant health risks for sponges (24). The researchers posed that LMA sponges with higher mortality than HMA sponges were accompanied by microbial community shifts and downregulation of host immune function under future 2100 climate conditions (26). Recent studies also suggested that HMA holobionts are characterized by stronger metabolic dependence and chemical defense (17). These imply that HMA and LMA sponges respond differently to the presence of environmental changes.

In a changing coastal environment, processes such as environmental factors, niche segmentation, and/or competition strongly influence community assembly and alternations (27). A description of the symbiotic microbial communities associated with sponges and their response to environmental factors that fluctuate greatly and regularly in nature could aid in understanding the coherence of the response of the holobiont to environmental stresses. To explore how the sponge-associated microbiome responds to environmental changes, we investigated the microbial community dynamics, including bacteria and archaea, in HMA sponge *Spongia officinalis* (Schulze, 1879) (class Demospongiae, order Dictyoceratida, family Spongiidae) and two LMA sponge species *Tedania* sp. (Gray, 1867) (class Demospongiae, order Poecilosclerida, family Tedaniidae) and *Haliclona simulans* (Johnston, 1842) (class Demospongiae, order Haplosclerida, family Chalinidae) in coastal zones of Fujian, China for 1 year. In addition, the microbiome of 13 other sponge species from the Sponge Microbiome Project (SMP) was also analyzed to elucidate potential and regular trends. Here, we attempt to decipher (i) the temporal variation in the sponge-associated microbial communities and correlations with environmental factors, (ii) the ecological functions of the sponge-associated microbial communities, and (iii) the adaptive rule of different sponges to dynamic environments.

## MATERIALS AND METHODS

### Sample collection and environmental factor measurements

Three dominant sponge species, *Tedania* sp., *Haliclona simulans,* and *Spongia officinalis,* were sampled at Zhao’an Bay, Zhangzhou City, Fujian Province, China (with geographic coordinates of 23.62°N and 117.34°E; Fig. S1) in 2022. Zhao’an Bay is located on the west coast of the Taiwan Strait and at the confluence of the East China Sea and the South China Sea, where the mariculture industry, which includes the confluence of sea and freshwater, has developed on a large scale. Sponges and ambient seawater were collected from different months (January, April, June, August, and October) in three replicates for 1 year. Individual surfaces of all sponge samples were rinsed with filtered seawater (0.22-µm filter), cut into 1 cm^3^ pieces with a sterile knife, and then temporarily transferred to 70% (vol/vol) ethanol on ice at −80°C in the laboratory. For the seawater sample, 300–500 mL of water was filtered through 0.22-µm polycarbonate filters (47 mm diameter; Millipore, Billerica, MA, USA). The filters were stored at −80°C until further processing. Seawater samples (three replicate individuals) for environmental factor measurement were collected at the same time and stored at −20°C until use.

Temperature, salinity, dissolved oxygen (DO), and pH were measured *in situ* with a portable multiparameter water quality tester (Multi 3510 IDS, WTW, Germany). Other environmental factors, such as nitrate (NO_3_^-^), nitrite (NO_2_^-^), dissolved inorganic phosphorus (DIP), and silicate (SiO_3_^2-^), were measured in the laboratory using an Auto Analyzer3 (AA3, Bran + Lubbe, D-22844, Norderstedt, Germany). Total phosphorus (TP) and total dissolved phosphorus (TDP) were measured by spectrophotometry after digestion (28). Dissolved inorganic phosphorus (DOP) was calculated as the difference between the TDP and DIP concentrations. The ammonium (NH_4_^+^) concentration was measured by the indophenol blue method (29).

### Electron microscopy

The fresh sponge tissue was rinsed with filtered seawater three times, cut into small strips of 2–3 mm cubic, and fixed with 2.5% glutaraldehyde/artificial seawater for 24 h to avoid cell deformation caused by osmotic pressure. *Tedania* sp. and *H. simulans* were immersed in hydrofluoric acid for half an hour to remove bone needles. *S. officinalis* has no spicules and is not processed. Subsequently, the three sponges were post-fixed in 2% OsO4 and dehydrated through a graded ethanol series (30%, 50%, 70%, 90%, 3 × 100%) for 15 min respectively, then the uranium saturated solution prepared with 70% ethanol and dyed. After the substitution of ethanol and acetone, the pieces were embedded in LR White resin for 24 h at 70°C. Ultrathin sections were stained with uranyl acetate and Reynolds lead citrate for 5 min, respectively. The resulting sections were photographed *via* transmission electron microscopy (TEM, HITACHI HT7800, Japan). Several different image examinations were performed on three biological specimens from each species.

### DNA extraction, PCR, and Illumina sequencing

Metagenomic DNA was extracted from sponges (*n* = 45, including three replicates of each sample) and seawater filters (*n* = 15) following the methods of Ou et al. (30). The V3-V4 region of the bacterial 16S rRNA genes was amplified using the primer sets 341F and 806R (31). The primers Arch519F and Arch915R (32) target the V4-V5 region of the 16S rRNA gene of archaea. The 25 µL PCRs involved initial denaturation at 98°C for 3 min; 30 cycles of 98°C for 10 s, 55°C for 20 s, and 72°C for 20 s; and a final extension at 72°C for 5 min. Triplicate PCR products for each sample were pooled and verified by 2% (wt/vol) agarose gel electrophoresis to confirm specificity. PCR products were purified and sequenced on the Illumina MiSeq PE300 platform [Sangon Biotech (Shanghai) Co., Ltd., China].

### Sequencing data processing

Chimeric sequences and monads were removed from raw data, and amplicon errors were corrected using the DADA2 package of R version 3.6.0 (33), and amplicon sequence variants (ASVs) were identified and tabulated. All sequences showed 100% similarity to the ASVs. After normalization of the data and archaeal sequences were removed, we obtained 568,000 high-quality bacterial sequences clustered into 2836 ASVs, and 222,000 high-quality archaeal sequences (removal of bacterial sequences) clustered into 711 ASVs. Our sequencing data showed that most of the microbial diversity had recovered (Fig. S2). Taxonomic ranks were assigned to ASVs using the SILVA ribosomal reference database release 132 (http://www.arb-silva.de/).

### Definition of abundant and rare taxa

In this study, all ASVs, including sponges and seawater samples, were classified into three categories (34, 35): abundant taxa (AT) were defined as ASVs with a relative abundance >1% in all samples, and ≥0.01% in partial samples but ≥1% in others; ASVs with a relative abundance <0.01% in all the samples, and <0.01% in partial samples but never ≥1% in any sample were defined as rare taxa (RT); ASVs with a relative abundance <0.01% in partial samples but ≥1% in others were defined as dominant taxa (DT). The detailed descriptions of the data sets used are presented in Table S1.

### Statistical analyses

#### Community diversity and structure

Alpha diversity indices were estimated in R version 3.6.0 with the vegan package (36). SPSS Statistics for Windows v.22.0 was used for one-way analysis of variance (ANOVA) to analyze the influence of temporal effects on alpha diversity. The bacterial and archaeal community compositions of the sponges were visualized using nonmetric multidimensional scaling (NMDS) based on Bray–Curtis dissimilarities (37) and were estimated using vegan (36). To investigate the differences between the microbial communities, analysis of similarity (ANOSIM), multiple response permutation procedure (MRPP), and permutational multivariate analysis of variance (PERMANOVA) were performed with the “vegan” R package (36). Linear discriminant analysis effect size (LEfSe) was calculated with LEfSe version 1.1.0. The set value was LDA score >2, less strict set to 2, and more strict was set as 4.

### Relationships between community composition and environmental variables

Canonical correspondence analysis (CCA) was used to analyze the relationships between microbial communities and environmental/spatial factors corresponding to the longest gradient lengths (>4 for all taxa in this study) of detrended correspondence analysis (DCA). Before the CCA, the environmental variables with a high variance inflation factor (VIF) >20 were eliminated to avoid the impact of collinearity among factors. In addition, Mantel tests were conducted to determine the relationships between bacterial community dissimilarity and environmental/spatial variables to determine the significance of the environmental factors. All computations were performed in the “vegan” R package (36).

### Niche width

The Levins coefficient was used to measure niche width (38):


(1) $${B_{i}=1/\sum }_{j=1}^{r}P_{ij}^{2}$$

In the above equation, Bi is the habitat niche width of ASV_i_, and P_ij_ is the proportion of ASV_i_ to total ASVs within a given resource state j. The niche width of the microbial community is subsequently expressed by averaging B across all ASVs.

### Network analysis

To analyze the interactions among microbial members, the Molecular Ecological Network Analyses Pipeline (MENAP) was used to construct molecular ecological networks (MENs) (39). All networks were constructed using more than 50% of the ASVs in all samples and constructed on the basis of Spearman correlations of non-log-transformed ASVs, followed by a random matrix theory-based (RMT-based) approach that automatically determined the correlation cutoff threshold. Mantel tests were performed between distance matrices of ASV connectivity (40).

To study the coexistence of microorganisms in sponges, seawater, and sponges, a bipartite network analysis was performed. We first assessed the inverse Simpson (InvSimpson, Fig. S3) index of microorganisms to determine the effective number of species (neff). Based on the recommendations of Weiss et al. (41) and evaluations of the performance of eight different methods (Bray–Curtis, Pearson, Spearman, CoNet, LSA, MIC, RMT, and SparCC), we decided to use the LSA method because our results do not conform to the high-diversity compositions with neff <13; moreover, the ASV table is less than 50% sparse, and our data are time series.

To examine the reliability of the constructed networks, 100 random networks were generated for each empirical network. The number of nodes and edges were fixed, and the links among the nodes were randomly rewired following the Maslov-Sneppen procedure (42). Next, the topological characteristics of both the empirical network and 100 random networks were calculated and compared. Network visualization was performed with Gephi version 0.10.1. PICRUSt2 was used to predict the functional characteristics of sponge microbial communities (43).

### Meta-analysis of sponge microbiome diversity and ecological functions

Selected data sets from the SMP (44) were retrieved from the Qiita database under Study ID: 10793 (March 2024). We selected sponge samples that were compliant with the following criteria: (i) from the same place and time, (ii) simultaneous presence of identified HMA and LMA sponges, (iii) samples of surrounding seawater were included in the database, (iv) were extracted from healthy, adult individuals, (v) at least three replicate samples where present in the data set, and (vi) these had  >10^4^ 16S rRNA gene sequencing reads. Only two sets of data viz. 42.05°N, 3.20°E (Northwest coast of the Mediterranean) and 21.43°N, 157.79°W (Hawaii, United States) (Fig. 6a) were eligible. Community diversity and structure, niche width, and network analysis were estimated as described above. At least six sponge samples have been subjected to construct MENs. Phylogenetic trees were constructed based on the 18S rRNA gene sequences of sponges by the neighbor-joining method with 1,000 bootstrap replicates using MAGA X 10.0.5.

## RESULTS

### HMA and LMA sponge classification

**Fig 1:**
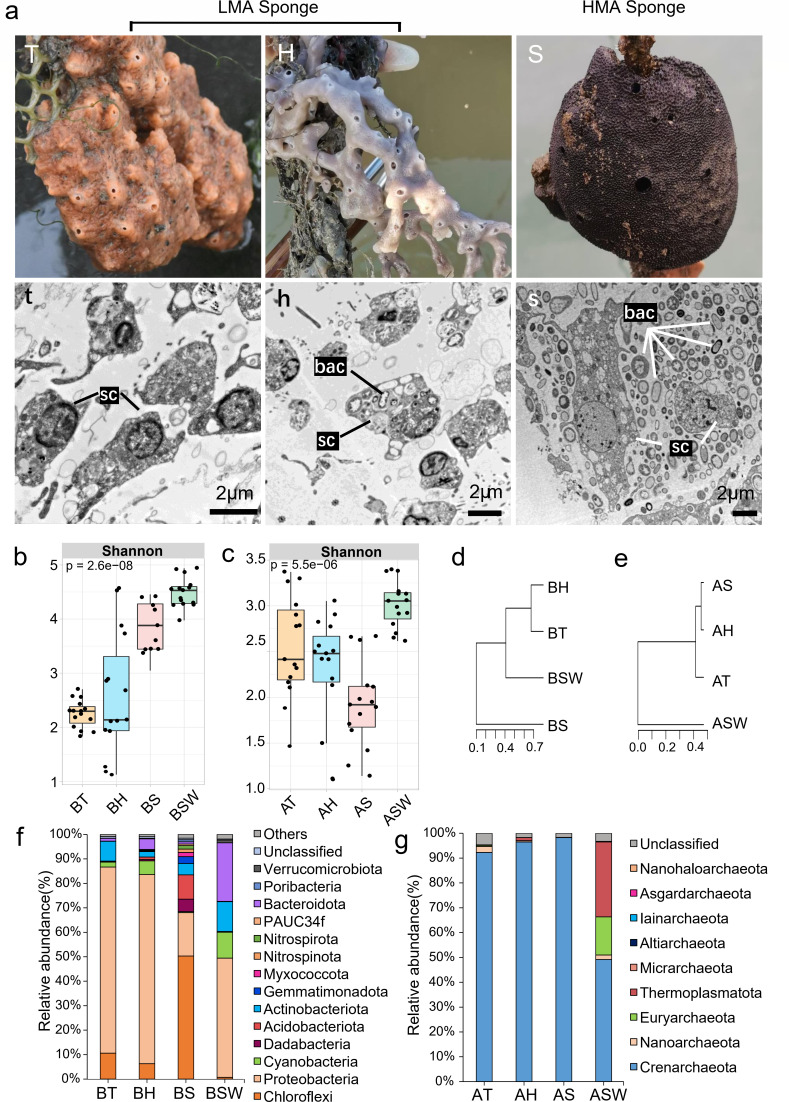
Differences between HMA and LMA sponges. (a) Pictures of three sponge species (**T, H, S**) and transmission electron microscopy observation (**T, H, S**). (b) Alpha diversity of the microbial community. (c) Microbial community composition (phylum level). (d) Hierarchical clustering dendrogram of microorganisms in sponge and seawater. The dendrogram was constructed based on the Bray–Curtis distance method. T = *Tedania* sp., H = *Haliclona simulans*, S = *Spongia officinalis*, SW = seawater, with “B” signifying bacteria and “A” signifying archaea; bac = bacteria; sc = sponge cells.

The HMA or LMA states of sponges were characterized by TEM observations (19) and host-associated microbial communities (18). The sponge *S. officinalis* was classified as an HMA sponge based on the presence of abundant and morphologically distinct microbial cells in the mesohyl according to multiple fields of TEM observation (Fig. 1a). *Tedania* sp. and *H. simulans* were defined as LMA sponges because their mesohyl sections were mostly devoid of microbial cells. The 16S rDNA sequencing results of the microbial communities further verified the above definition that the HMA sponge harbored more diverse bacteria than did the LMA sponges (Fig. 1b). In the HMA sponge *S. officinalis*, phyla such as Chloroflexi, Acidobacteria, Dadabacteria, and Gemmatimonadetes of the bacterial kingdom were more abundant than were *Tedania* sp. and *H. simulans* (Fig. 1f). Associated with the sponge *Tedania* sp., the most abundant bacterial phyla were Proteobacteria and Actinobacteria, while the sponge *H. simulans* was harbored by more rich phyla, such as Proteobacteria, Cyanobacteria, and Bacteroidetes. These results were similar to previously reported microbial communities in HMA and LMA sponges (18). However, in the Archaea kingdom, the phylum Crenarchaeota (92.27% in *Tedania* sp., 96.47% in *H. simulans,* and 98.27% in *S. officinalis*) dominated in all sponges detected in this study, and no obvious differences were detected between sponges (Fig. 1g). We noticed that the bacterial clusters associated with LMA sponges were more closely related to seawater-derived taxa, but the similar result was not found in the archaeal community of the sponges (Fig. 1d and e). In addition, we also observed significant differences in the biomarkers of the bacterial community between sponges and seawater, while no differences were found in the archaeal community of sponges (Fig. S4).

### Fluctuations in the sponge-associated microbial communities during seasonal changes

**Fig 2:**
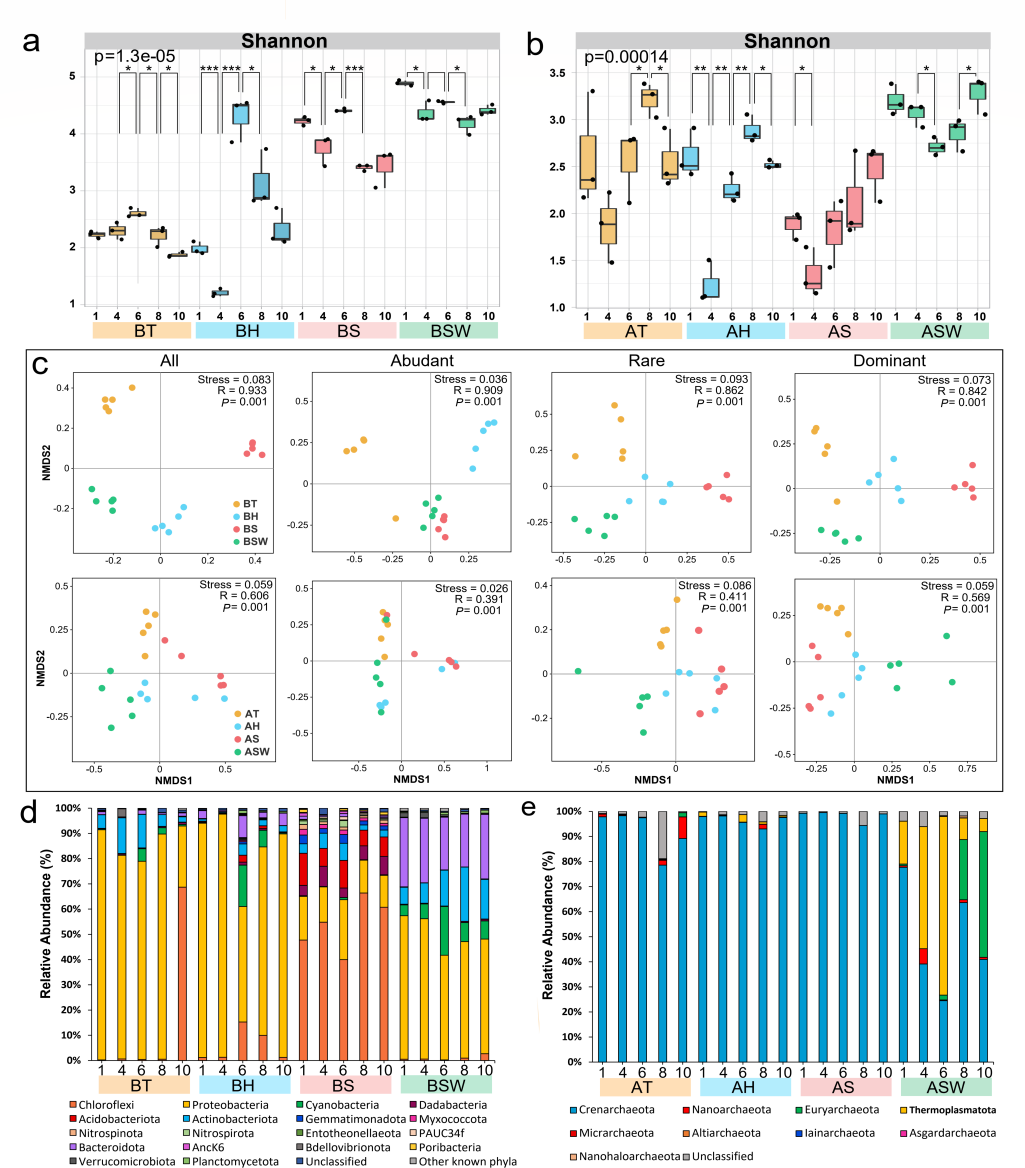
Diversity and composition of microbial communities in sponges and seawater. (a and b) Alpha diversity (Shannon index) of the microbial community. (c) NMDS ordination of sponge and seawater microbial communities based on Bray-Curtis distances. (d and e) Microbial community composition (phylum level) under seasonal variation. The Arabic numerals in the x-axis represent different months in 1 year. Data = mean (*n* = 3). A significance of alpha diversity in different months is shown: *P < 0.05, **P < 0.01, ***P < 0.001. T = *Tedania* sp., H = *Haliclona simulans*, S = *Spongia officinalis*, SW = seawater, with “B” signifying bacteria and “A” signifying archaea.

The microbial alpha diversities of the sponges and seawater were significantly different and changed over time (Table S4). The alpha diversity of the seawater bacterial and archaeal communities was greater than that of the sponges throughout the year (Fig. 2a). The average Shannon diversity of the bacterial communities from HMA sponges (*S. officinalis*, 3.84) was greater than that of the LMA sponges (*Tedania* sp.*,* 2.24 and *H. simulans*, 2.59). By contrast, the average Shannon diversity of the archaeal communities from *S. officinalis* (1.94) was lower than that of *Tedania* sp. (2.58) and *H. simulans* (2.31). From April to June, microbial Shannon diversity increased as temperatures rose. Among bacterial communities, Shannon diversity peaked in June and decreased significantly in August. However, the diversity of sponge-associated archaeal communities consistently increased from June to August. On the whole, the variation in bacterial communities was greater than that in archaeal diversity. In addition, the alpha diversity of the abundant, dominant, and rare taxa also exhibited similar temporal patterns in the sponges and seawater (Table S2; Fig. S3).

The microbial communities within the sponges and seawater were significantly separated according to NMDS and ANOSIM, and this separation was also reflected in the abundant, rare, and dominant taxa (Fig. 2b; Table S3). In addition, the bacterial community composition (*R* > 0.842, *P* = 0.001) was more distinct than the archaeal community composition (*R* > 0.391, *P* = 0.001). The rare taxa showed the most clear temporal variations in sponges and seawater (Table S3). More noticeable changes in bacterial communities over time were found in *Tedania* sp. and *H. simulans* than in *S. officinalis* (Fig. 2b; Table S3). Compared with those in bacteria, the archaeal community in *Tedania* sp. demonstrated smaller temporal variations than *H. simulans* and *S. officinalis*.

The microbial composition within the sponges and seawater also exhibited significant differences and temporal dynamics (Fig. 2c; Fig. S5). The microbial composition of *S. officinalis* was relatively stable with respect to temperature variation. However, for *Tedania* sp., the relative abundance of Chloroflexi increased sharply in October compared with August (Fig. 2c). Compared with those in April, the relative abundances of Cyanobacteria, Bacteroidota, and Chloroflexi in *H. simulans* increased in June. The archaeal communities associated with the sponges exhibited little fluctuation among the different seasons, and Crenarchaeota was the predominant and most stable community among the sponges. Only the relative abundance of Nanoarchaeota within *Tedania* sp. increased in October compared with August. Through further analysis, a marked increase of *Pseudohongiella* sp. in *Tedania* sp. and *H. simulans* was found in August (Fig. S5c). Further analysis revealed that *Tedania* sp. and *H. simulans* exhibited temporal variation, while *S. officinalis* exhibited relatively stable microbial communities among all the taxa (Fig. S6).

### Effects of environmental factors on sponge-associated microbial communities

**Fig 3:**
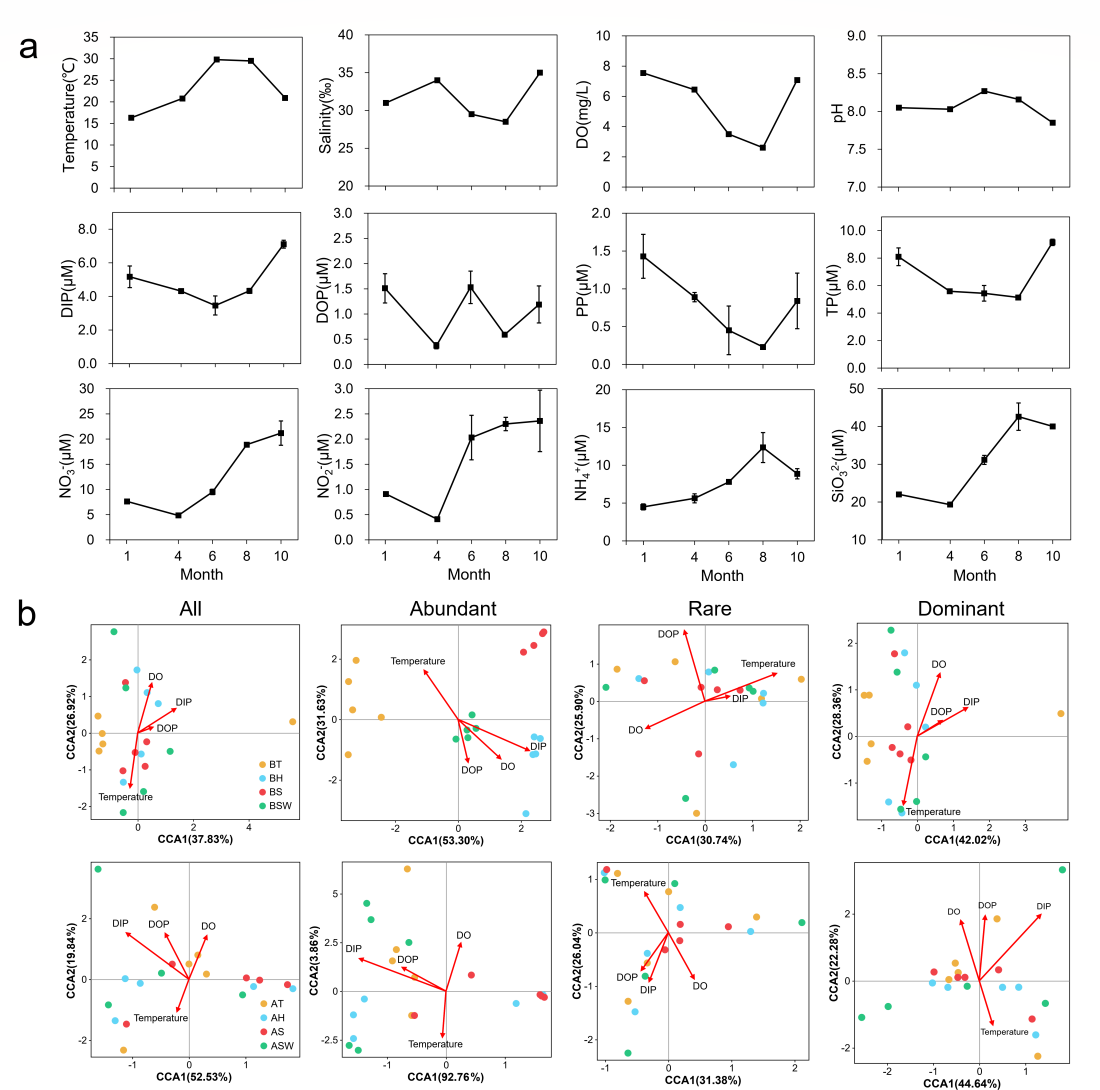
Microbial community structure and its correlation with environmental factors. (a) Dynamics of seawater environmental parameters during the study period. CCA was used to analyze the relationships between microbial communities and environmental factors and between different taxa (b). T = *Tedania* sp., H = *Haliclona simulans*, S = *Spongia officinalis*, SW = seawater, with “B” signifying bacteria and “A” signifying archaea.

Although limited samples for environmental factor measurements could not accurately describe the changes in coastal environments, the results exhibited notable variation tendencies over 1 year (Fig. 3a; Fig. S7). The microbial communities associated with sponges and seawater exhibited different responses to environmental factors over time (*P* < 0.05; Table S4). All the microbial communities in the seawater were more susceptible than those in the sponges to environmental influences. The CCA analysis indicated that temperature, DO, DIP, and DOP were the key factors influencing microbial community stability at all taxonomic levels, including all, abundant, dominant, and rare taxa (Fig. 3b). Among them, temperature had the most significant effect on the bacterial communities. In particular, for the bacterial communities, the abundant taxa within all sponges exhibited consistent temporal variation, whereas the rare taxa were relatively more affected by environmental factors (Fig. 3b; Table S4). A comprehensive comparison revealed that the HMA sponge *S. officinalis* were less subject to fluctuations in environmental factors than were those of the LMA sponges and seawater. In terms of the microorganisms, among the top 15 ASVs significantly associated with physicochemical factors during the course of 1 year (Fig. S7; Table S5), including Proteobacteria and Chloroflexi in *Tedania sp*. and *H. simulans*, and Chloroflexi and Acidobacteriota in *S. officinalis*. Among the archaeal communities, Crenarchaeota exhibited significant environmental correlations among all the sponge samples (Table S5).

Using bipartite networks, we observed for both ASVs from sponges or seawater, the cooperation and mutual benefits of microbial members were more predominant than the antagonistic effects (Fig. S8; Table S6). On average, the negative effects of microorganisms between sponges and seawater were more significant than the positive effects. In addition, the influence of seawater microbes, especially the Proteobacteria and Bacteroides, on bacterial communities was stronger than that on archaeal communities in sponges (Table S7). Seawater microbes had little effect on sponge archaea.

### Interspecific interactions of sponge-associated microbial communities

**Fig 4:**
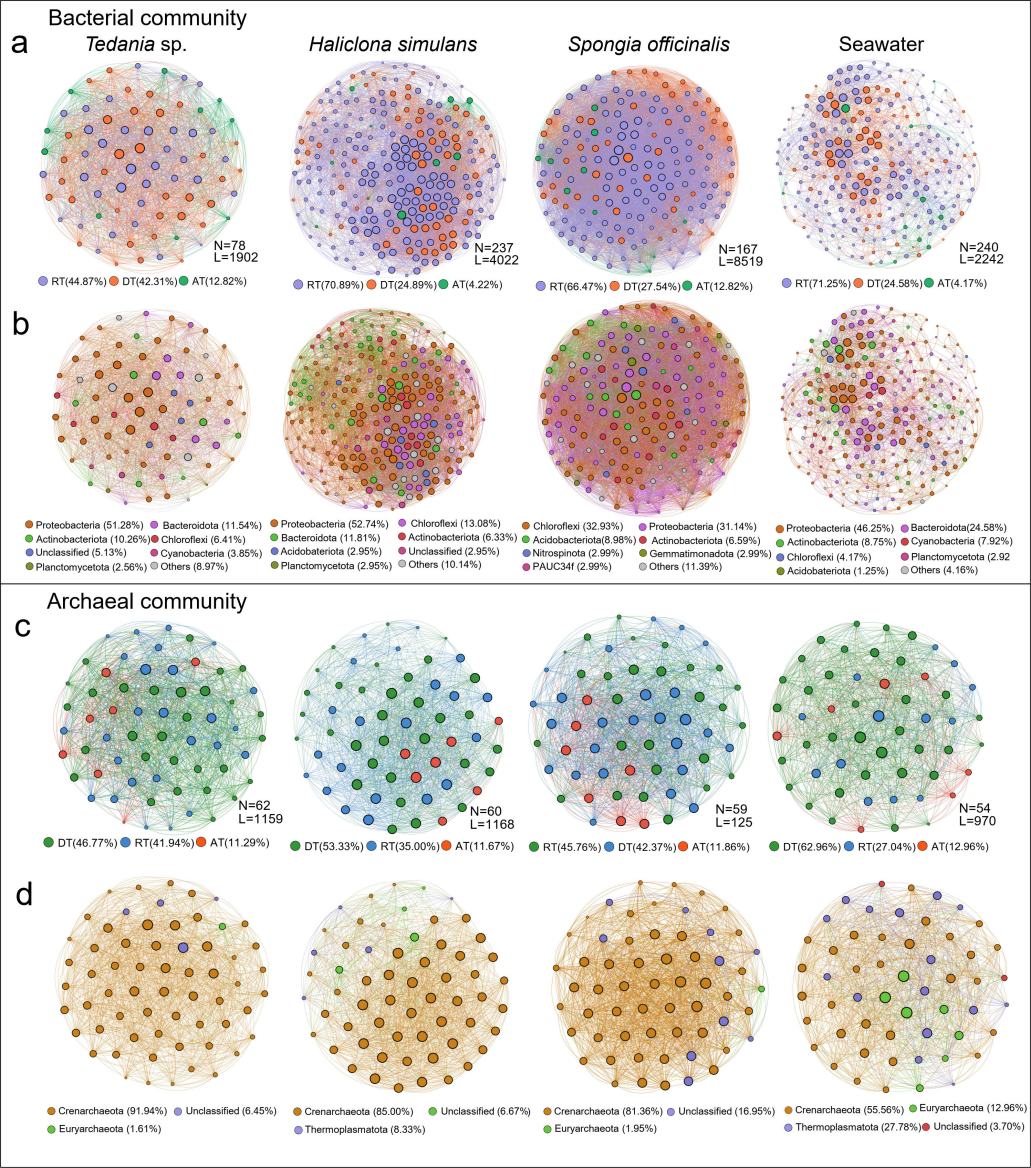
Properties of the correlation-based bacterial (a and b) and archaeal (c and d) networks. Network analysis shows the intra-associations within each subcommunity and inter-associations between different subcommunities. The size of each node is proportional to the number of connections (i.e., degree). The nodes are colored according to different types of taxa (a and c) and microbial community compositions at the phylum level (b and d). The numbers in brackets represent the percentage of nodes in different categories. N nodes, L links, AT abundant taxa, RT rare taxa, DT dominant taxa. T = *Tedania* sp., H = *Haliclona simulans*, S = *Spongia officinalis*, SW = seawater, with “B” signifying bacteria and “A” signifying archaea.

MENs were built based on the correlation relationships, and they indicated that the bacterial communities were more complex than the archaeal communities (Fig. 4; Table S8). In both the bacterial and archaeal networks, the microbial network relatedness was relatively greater in the sponges than in the seawater, implying that more dispersed microbial populations occurred in seawater (Table S8). Regardless of the presence of bacteria or archaea, the average connectivity (avgK) of the HMA sponge (*S. officinalis*, 102.02 for bacteria, 42.44 for archaea) was significantly greater than that of the LMA sponges *Tedania* sp. (48.77 for bacteria, 37.39 for archaea) and *H. simulans* (57.75 for bacteria, 38.93 for archaea), suggesting that the HMA sponges had greater microbial network complexity and tighter relationships in a dynamic environment. In addition, rare taxa accounted for 44.87%–71.25% of the bacterial networks and 24.07%–45.76% of the archaeal networks and implemented multilateral roles in the microbial network.

Chloroflexi, Proteobacteria, Actinobacteria, Acidobacteria, and Crenarchaeota were composed of major keystone species that contributed to sponge species specificity. According to the average degree and betweenness centrality, a total of the top 10 microbial ASVs were defined as keystone species of each sample, and they were considered dominant or rare taxa (Fig. 4a and c; Table S9). Among them, Crenarchaeota was the keystone species in all sponges. It was evident that sponges had unique microbial communities despite being influenced by environmental factors (Table S10). In addition, according to the bipartite network of bacterial and archaeal communities (Fig. S9), the proportions of bacteria and archaea in *Tedania* sp. were similar in the microbial bipartite network, while *H. simulans* and *S. officinalis* were dominated by bacteria, accounting for 74.63% and 77.17%, respectively.

### Differences in ecological functions of different sponges

**Fig 5:**
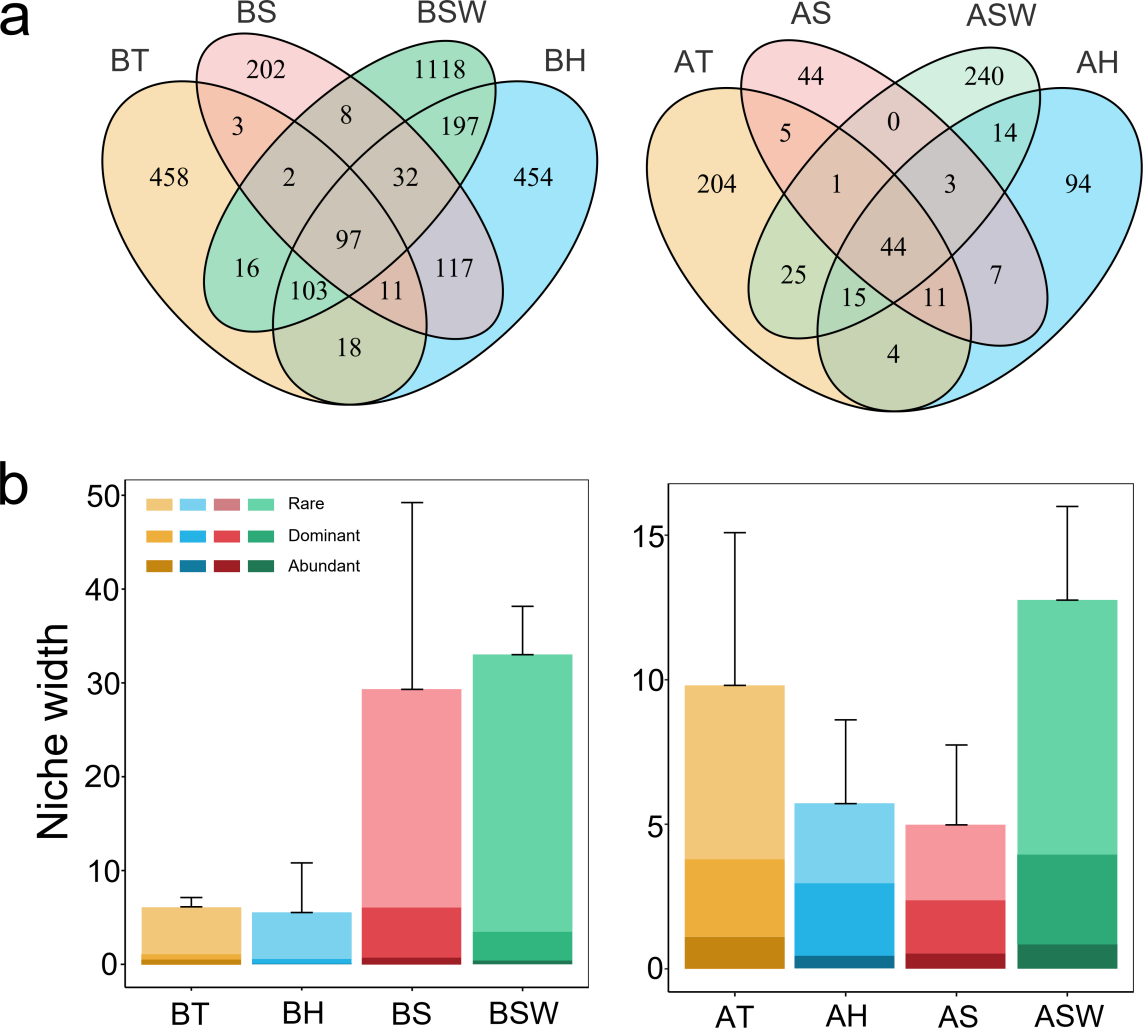
Ecological functions of sponges. (a) Venn diagram showing the numbers of unique and shared ASVs in the microbial community between sponges and seawater. (b) The niche width of the sponges and seawater microbial communities. Values = mean ± standard error, *n* = 3. T = *Tedania* sp., H = *Haliclona simulans*, S = *Spongia officinalis*, SW = seawater, with “B” signifying bacteria and “A” signifying archaea.

Venn diagram analysis revealed that the three sponges shared significantly different microbes with seawater. Among them, *Tedania* sp., *H. simulans,* and *S. officinalis* shared 30.79% (218/708), 41.69% (429/1,029), and 29.45% (139/472) of the bacteria, respectively, with seawater, while 27.51% (85/309), 39.58% (76/192), and 41.74% (48/115) of the archaea were ubiquitous with seawater (Fig. 5a). The results also showed the presence of specific or shared ASVs in the microbial community of all the samples, including the abundant, rare, and dominant taxa (Fig. S10). Notably, for both bacteria and archaea, the specific microbes in sponge hosts and seawater were mainly rare taxa (Fig. S10).

Niche width is an indicator of the diversity of resources used by organisms (45). To investigate the role of microbes in different sponges, we calculated the average niche width of the microbial community. Overall, seawater-derived microbes had a relatively greater niche width than all sponges (Fig. 5b). The average niche width of the bacterial communities was much greater in *S. officinalis* (29.04) than in *Tedania sp*. (6.05) and *H. simulans* (5.45). In the archaeal community, *Tedania* sp. had the highest average niche width, while *S. officinalis* had the lowest. Comparing all the taxa, we also noted that the niche width of rare taxa in both the bacterial and archaeal kingdoms had the highest value, indicating greater biological resource utilization. Using PICTRUs2 analysis, we found significant differences in metabolic functions between sponges and seawater. Interestingly, the metabolic functions of the archaeal groups in sponges were quite similar but were obviously different from those of the archaeal groups in seawater (Fig. S11).

### Microbiome structure and ecological functions of diverse sponge species

**Fig 6:**
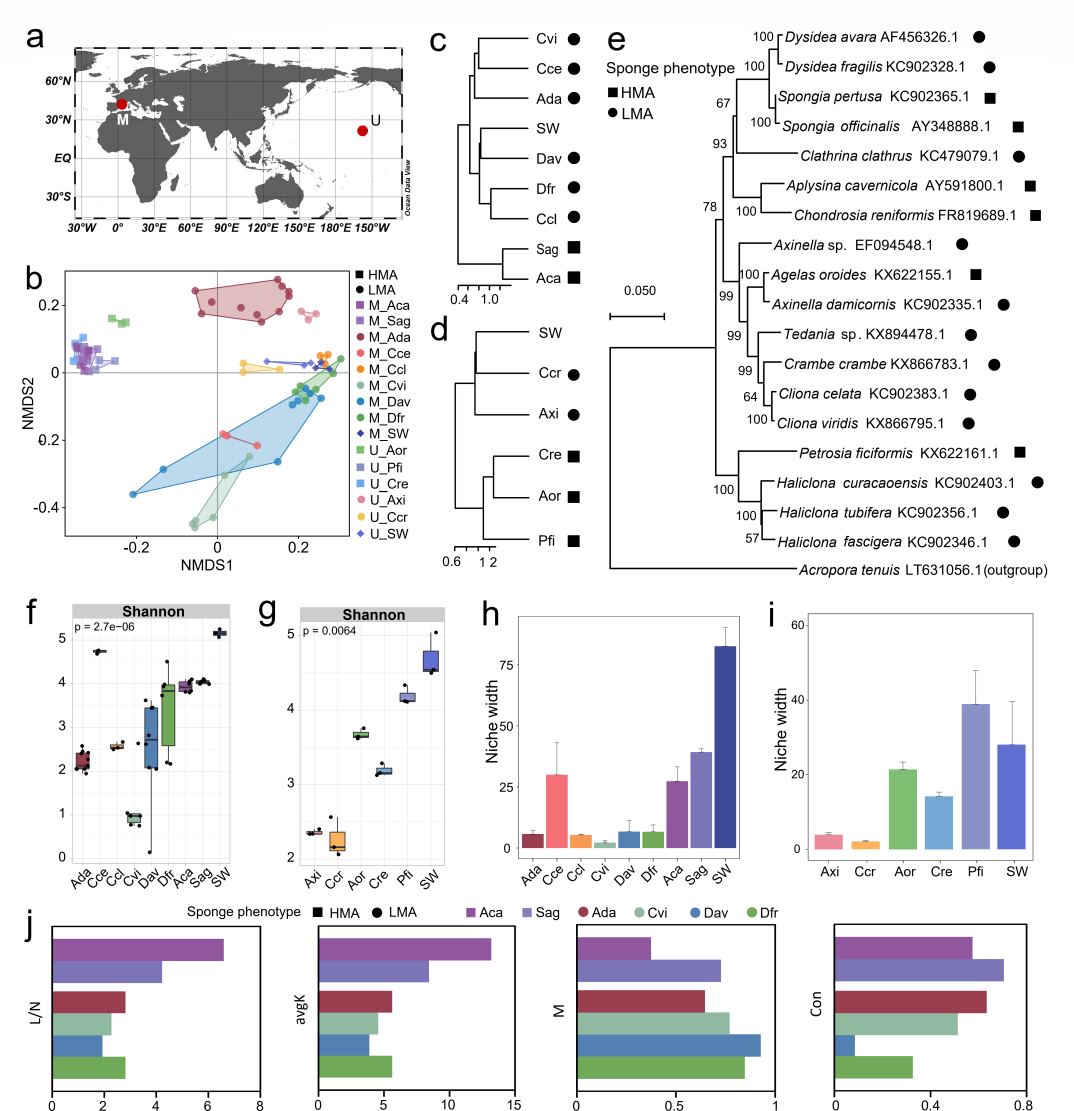
Microbiome diversity and ecological functions of diverse sponge species. (a) Map of sampling sites retrieved from the Sponge Microbiome Project. The map was drawn by Ocean Data View (ODV) version 5.2.0 (46). M northwest coast of the Mediterranean (42.05°N, 3.20°E, 2007), Hawaii, United States (21.43°N, 157.79°W, 2012). (b) NMDS ordination of microbial communities based on Bray-Curtis distances. (c and d) Hierarchical clustering dendrogram of sponge adults and seawater at the M (c) and U (d) stations retrieved from the Sponge Microbiome Project. The dendrogram was constructed based on the Bray–Curtis distance method. Sponge phenotype is indicated by shapes next to the species name. (e) Phylogenetic tree of the sponge 18S rRNA gene sequences constructed with the neighbor-joining method. The 18S rRNA gene sequence of *H. simulans* was not queried by the National Center for Biotechnology Information (NCBI) and was replaced by three sponges of the same genus. (f and g) Alpha diversity (Shannon index) of bacterial communities at the M (f) and U (g) stations. (h and i) The niche width of the sponges and seawater microbial communities at the M (h) and U (i) stations. Values = mean ± standard error, *n* ≥ 3. (j) topological characteristics of the MENs of diverse sponge-associated microorganisms, including links/nodes (L/N), average connectivity (agvK), Modularity (**M**) and Connectedness (Con). *Aplysina cavernicola* (Aca) (*n* = 6), *Axinella damicornis* (Ada) (*n* = 12), *Cliona celata* (Cce) (*n* = 3), *Clathrina clathrus* (Ccl) (*n* = 3), *Cliona viridis* (Cvi) (*n* = 6), *Dysidea avara* (Dav) (*n* = 11), *Dysidea fragilis* (Dfr) (*n* = 6), and *Spongia agaricina* (Sag) (*n* = 6) come from northwest coast of the Mediterranean. *Axinella* sp. (Axi), *Agelas oroides* (Aor), *Crambe crambe* (Ccr), *Chondrosia reniformis* (Cre), *Petrosia ficiformis* (Pfi) come from Hawaii, United States, and three replicate samples in all sponge species. *A. oroides* and *S. agaricina* were defined as HMA sponge, and *A. damicornis*, *C. celata*, *C. viridis*, *C. clathrus*, *D. avara*, *D. fragilis,* and *Axinella* sp. were defined as LMA sponge were according to Moitinho-Silva et al. (18). *A. cavernicola*, *C. reniformis,* and *P. ficiformis* were defined as HMA sponge and *C. crambe* was defined as LMA sponge were according to Gloeckner et al. (19).

NMDS analysis showed the symbiotic bacterial communities of HMA and LMA sponges were clearly separated from each other (Fig. 6b). And the LMA sponge-associated bacterial communities were more similar to seawater than those associated with HMA sponges. Individual differences were far lower in HMA sponge symbionts than in LMA sponges. Significantly, HMA sponge-associated bacterial community composition was more similar beyond the divergences in species, sampling sites, and time than those from the LMA sponge (Fig. 6a and b). The similar results were observed in hierarchical clustering (Fig. 6c and d), even though these sponges clustered within different lineages (Fig. 6e). Except for *Cliona celata* (Cce) and part of the *Dysidea fragilis* (Dfr) samples, all HMA sponges had higher bacterial diversity than LMA sponges (Fig. 6f and g). The average niche width (29.74) of the five sponges defined as HMA sponges was greater than that of the eight LMA sponges (6.86) (Fig. 6h and i). Surprisingly, *C. celata*, defined as LMA sponges, showed distinctiveness, exhibiting similar microbial diversity and niche width to HMA sponges.

Only six sponge samples were constructed for MENs, of which two were HMA sponges and four were LMA sponges. The network complexity of different sponge-associated bacteria was compared according to the network topology parameters. Overall, the links/nodes (L/N), avgK, Modularity (M), and Connectedness (Con) parameters all showed that HMA sponges *Aplysina cavernicola* (Aca) and *Spongia agaricina* (Sag) were more complex than LMA sponges (Fig. 6j; Table S11; Fig. S12).

## DISCUSSION

Sponges harbor complex microbial communities that not only help hosts to provide ecosystem services and support biodiverse but also increase the flexibility of the host under environmental stress (13, 47). Here, we investigated the temporal community characteristics of microbial symbionts that maintained a unique microbial network structure and homeostasis in three sponge species. Meta-analysis of microbial communities of 13 sponges belonging to 11 genera from SMP was performed to further elucidate the regular adaptation.

### Sponge-specific and species-specific symbiont associations

The host specificity of sponges is associated with an extremely diverse symbiont community (8, 48). This was reflected in the microbiological compositions and NMDS analysis of the sponge microbiome in this study. On the other hand, the significant differences in microbial communities among various sponge species could be driven by differences in symbiosis status (i.e., the HMA–LMA dichotomy) rather than geographic location. The same results have been confirmed for marine sponge species both at large spatial scales across the Caribbean (15) and at small spatial scales within coral reef systems (49). The HMA sponge had a greater abundance of Chloroflexi, Gemmatimonadetes, Poribacteria, and Dadabacteria strains (50–52) that conferred greater environmental adaptability, metabolic dependence, and chemical defense onto HMA holobiont in dynamic environments (17).

In contrast to the symbiotic bacteria, archaea associated with sponges have been less studied and exhibit low abundance (53, 54). We did not find a clear HMA–LMA dichotomy in archaeal communities. All sponges possessed the symbiont *Candidatus Nitrosopumilus* [Crenarchaeota, now affiliated with the phylum Nitrososphaerota (55)], which could play an important role in ammonia oxidation (56) and appeared to be a strict symbiont of sponges. Therefore, the representative microbial group for the HMA–LMA dichotomy was bacteria with higher abundance and diversity (57). Further analyzing the differences in microbial taxa, we noted that the rare microorganisms associated with ASVs accounted for more than 90% of the bacterial community and 80% of the archaeal community in all the samples. It implies that microorganisms enter the rare biosphere and exhibit host specificity in both HMA and LMA sponges (58, 59).

### Environmental factors drive microbial community shift

Symbiotic microbial communities could change with physical and chemical parameters in the elastic range (60). Mantel tests showed that the microbial communities in sponges were less affected by environmental factors than were those in seawater, suggesting that sponges have become stable “containers” for microorganisms (8). The diversity of sponge symbiotic microbes increased in April and reached its peak in June, but the diversity decreased significantly in August. Appropriate warming increases microbial diversity, as does the planktonic marine bacteria (61), but long-term high temperatures actually reduce microbial diversity (62). In addition, a degree of hypoxia increases microbial diversity (63), but the DO concentration lower than what is needed for sponges results in a reduction in microbial diversity, as in anoxic basin waters (64). In the future 2100, the excessive temperature will be accompanied by a decrease in DO that may lead to the sharp decline of sponge bacterial diversity or even the collapse of a bacterial network (24, 65). Interestingly, during sustained high temperatures (~30°C) from June to August, the diversity of archaeal communities increased in sponges. In the most extreme environments, there is already evidence for the predominance of archaea (66). We speculated that the sponge-associated microbiome may gradually a transition from a bacteria-dominated to an archaea-dominated symbiotic microbial system in future environmental conditions.

Symbionts qualify as sponges with elasticity to an alternative stable state after a disturbance (5). A comparable analysis indicated that the microbial community composition stability of *Tedania sp*. and *H. simulans* was less than that of *S. officinalis*. In particular, in *Tedania* sp., the abundance of the SAR202 clade (Chloroflexi, Dehalococcoidia) and Nanoarchaeota clearly increased in October. SAR202 accumulates genes for degrading stubborn organic matter of origin (67). The large input of terrigenous organic matter could lead to more SAR202 enrichment in *Tedania* sp. inhabiting the coastal areas. In addition, the increase in the abundance of Nanoarchaeota in October could support the adaptability of symbionts to low-pH environments (68). With high ammonia stress, depletion of oxygen, and high PH occurred in August, there was a marked increase in *Pseudohongiella* sp. in *Tedania* sp. and *H. simulans*, which could be one important indicator for how microbiome reacts to their environment, even though no obvious variation was found at the phylum level. It has been reported that *Pseudohongiella* sp. is liable to integrate foreign DNA into their genomes through horizontal gene transferring, enhancing the capacity of the metabolism of certain substrates and the response to environmental changes (69). However, *S. officinalis* (classified as the HMA sponge) showed the stability of the microbial community and resistance to environmental changes.

### Potential roles of seawater microbes on sponge symbionts

The distinction between “resident” sponge symbionts and “transient” seawater bacteria in sponges has been widely discussed (16). The patterns of interactions among these microorganisms through bipartite networks showed that the microorganisms from each sponge and seawater were independent but closely related. The composition of the bacterial community in seawater remained relatively stable throughout the year, while sponge-associated bacterial flora exhibited seasonal variation. All sponges studied here shared nonnegligible bacterial ASVs with seawater, especially *Tedania sp*. and *H. simulans*, whose microbial community composition was similar to seawater, speculating that a large proportion of symbionts may be affected by horizontal transmission. Recent studies support that environmental acquisition is a major contributor to the sponge microbiome (70). The “sponge microbial specificity” alludes to the primary role of microbes in vertical transmission (16, 71). After several generations of hosts, long-distance diffusion of offspring can show the defects of vertical propagation (16). Leaky vertical transmission is principally vertical with occasional environmental acquisition, or vertical with massive environmental swamping (72). Host that exhibits varying degrees of leakage in vertical propagation of host species may reflect different evolutionary solutions (72). It has been shown previously that strict vertical transmission patterns are not observed in sponges (73). This leaky vertical transmission mode of sponges increases genetic diversity, which balances the shortcomings of pure vertical or horizontal propagation models. It has been reported that LMA sponges retain beneficial symbionts from seawater to cope with environmental changes through more horizontal transfer (70), implying a symbiont strain that spreads through a particular vertical may not be optimal in all habitats. HMA sponges strengthen the connection between the host and symbiont through more vertical transmission, although also still exist environmental transfer, allowing the “container” to maintain a relatively independent and intact microbial community to adapt to environmental stress (16, 71). Clearly, different sponge species have exhibited the optimal mode of symbiotic transmission in response to environmental changes.

### Assembly and ecological function of sponge-associated microorganisms in dynamic environments

In the present study, MENs exhibited significant host specificity in microbial assembly, including the rare, abundant, and dominant taxa in sponges. *S. officinalis* had a stronger inter-microbial association than those of *Tedania sp*. and *H. simulans*, which might be related to the differences in nutrient metabolism of the host and in function-specific selection for different microorganisms (13, 15). Several lines of evidence suggest that microbial selection within sponges should be driven by factors beyond stochasticity or environmental filtering provided by the water column (74). The evolutionary origin of specific symbiotic microbes may be the main driving force in constructing host-associated microbiomes.

According to PICTRUs2 and NMDS analyses, sponge species specificity and microbial community exclusivity may contribute to their different ecological functions. The difference among *S. officinalis*, *Tedania sp*., and *H. simulans* was further illustrated in the diversity of biological utilization resources, which reflected the ability of species or populations to adapt to the environment (45). A wider niche predicted that the species were less specialized and that the competition for resources was more intense. Therefore, the bacterial communities of *S. officinalis* could use various resources more efficiently than *Tedania sp*. and *H. simulans* could, whereas in archaeal communities, *Tedania sp*. and *H. simulans* seemed more efficient, suggesting that the different kinds of sponge (i.e., HMA and LMA sponge) have different patterns for adapting to coastal zones.

### Sponge-associated microorganisms show different characteristics to adapt to the environment

**Fig 7:**
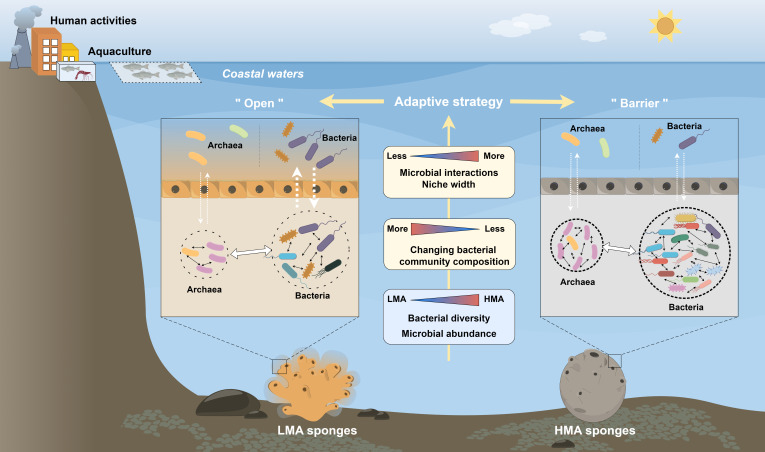
HMA and LMA sponges have different adaptation strategies to cope with environmental changes. The symbiotic microbial communities of HMA sponges were highly abundant and diverse, had tight microbial association, maintained a stable microbial community composition, and had higher ecological resource utilization capacity. Therefore, it adopted a barrier-like adaptation strategy to cope with the variable environment. On the other hand, although the microbial diversity and abundance of LMA sponges were lower than those of HMA sponges, LMA sponges could adapt to environmental stress by more significant horizontal transmission, loose structure of the microbial community, and changing their microbial community composition, forming an open-like adaptation strategy. The drawing was done by Figdraw.

Meta-analysis showed that quite different microbial communities and ecological functions between HMA and LMA states were attributed to trophic ecology patterns of sponges. Microorganisms account for the majority of DOM uptake by HMA sponges, but are mainly dependent on the host for nutrients in LMA sponges (75). Denser mesohyl and slower filtration rates commonly occur in HMA species but the opposite happens in LMA sponges (21). This may be related to the fact that LMA holobiont with lower tissue density and higher pumping rates experienced more frequent seawater exchange and more environmental communication. Therefore, the vulnerability to environmental perturbations could be weakened by altering microbial communities or accumulating beneficial microorganisms from the ambient seawater. It was previously reported that sponges descended from the LMA symbiotic state (17). During a long evolution, HMA sponges could be developed by enhancing chemical defenses and reducing the palatability of sponge species (17). The formation of phenotype may be driven by ecologically specialized selection pressures in which HMA sponges harbor more diverse and specialized microbial communities to acclimatize environments (17). HMA symbionts continuously evolve by recruiting similar combinations, which beyond temporal, geographic, and species divergence, show stronger evolutionary signals than LMA sponges. We proposed that sponge-associated microbiomes developed different adaptative strategies to the environment: HMA sponge-associated microbiomes were relatively closed with higher diversity, stronger stability, and closer interactions which could be in a protected state like a “barrier,” while LMA was relatively more dependent on horizontal transmission and more affected by environmental conditions which could be in an open way (Fig. 7). The conception contributes to the understanding the successful evolution of HMA and LMA sponges and helps to predict their future development trends in response to future global changes.

### Conclusion

Our study provided a more comprehensive understanding of the highly diverse, host specific, and niche-divergent symbiont microorganisms associated with different sponge species in coastal zones. We observed the HMA–LMA dichotomy only in sponge-associated bacteria. HMA and LMA sponges are considered two basic life strategies that evolved and exhibited contrasting characteristics in dynamic environments. Bacteria associated with the HMA sponges *S. officinalis* exhibited a broader niche and tight-connected community with high diversity and strong stability to challenge the influence of anthropogenic, while *Tedania* sp. and *H. simulans* assigned into LMA appeared to be more willing to integrate into the environment and adopted a loose-connected community to adapt to dynamic environments. A meta-analysis from 13 sponges also supported this concept. In addition, HMA symbionts are highly similar beyond temporal, geographic, and species divergence. Our results also implied the ecological functions of archaea are higher in LMA sponges than in HMA sponges and sponge-associated archaea may have a dominant position in the future conditions and support for “LMA sponge ancestor.” Symbiotic microorganisms are important avenues for reflecting differences in host evolution and ecological functions; however, more research is necessary to understand the co-evolution of HMA and LMA symbionts and hosts in symbiotic systems. Convergent evolution of hosts and symbionts may be a successful manifestation of holobionts adapting to different environments.

## Data Availability

Raw sequences were deposited in the Sequence Read Archive (SRA) operated by the National Center for Biotechnology Information (NCBI) under BioProject accession number: PRJNA1052941.

## SUPPLEMENTAL MATERIAL

Supplemental figures spectrum.01448-24-s0001.docx Fig. S1 to S12.

Supplemental tables spectrum.01448-24-s0002.xlsx Tables S1 to S11.

ASM does not own the copyrights to Supplemental Material that may be linked to, or accessed through, an article. The authors have granted ASM a non-exclusive, world-wide license to publish the Supplemental Material files. Please contact the corresponding author directly for reuse.
