# Supplementary material for: Dynamic microbiome diversity shaping the adaptation of sponge holobionts in coastal waters
Source: Microbiol Spectr. 2024 Oct 14;12(11):e01448-24. doi: 10.1128/spectrum.01448-24 (PMC11537060; doi:10.1128/spectrum.01448-24)
Supplement: Supplemental figures — Fig. S1 to S12. [file spectrum.01448-24-s0001.docx]

**Dynamic microbiome diversity shaping the adaptation of sponge holobionts in coastal waters**

**Supplementary Figures**


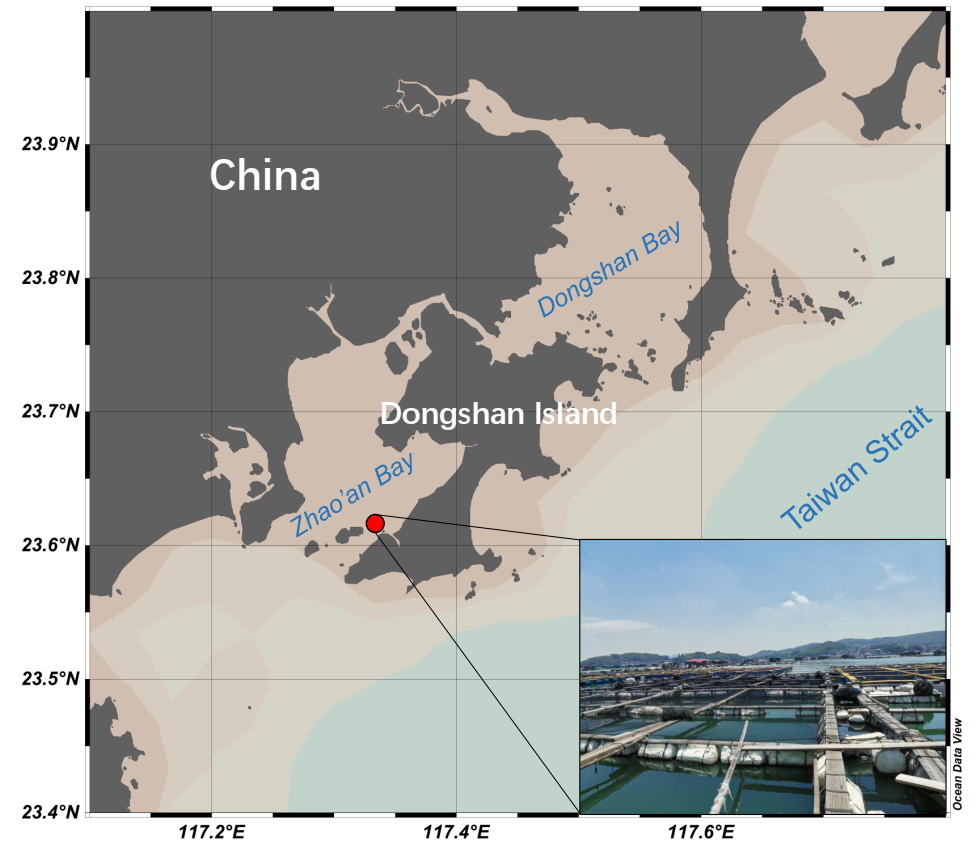


Fig. S1 Map of sampling sites in coastal aquaculture zones. The map was drawn by Ocean Data View (ODV) version 5.2.0 (1).


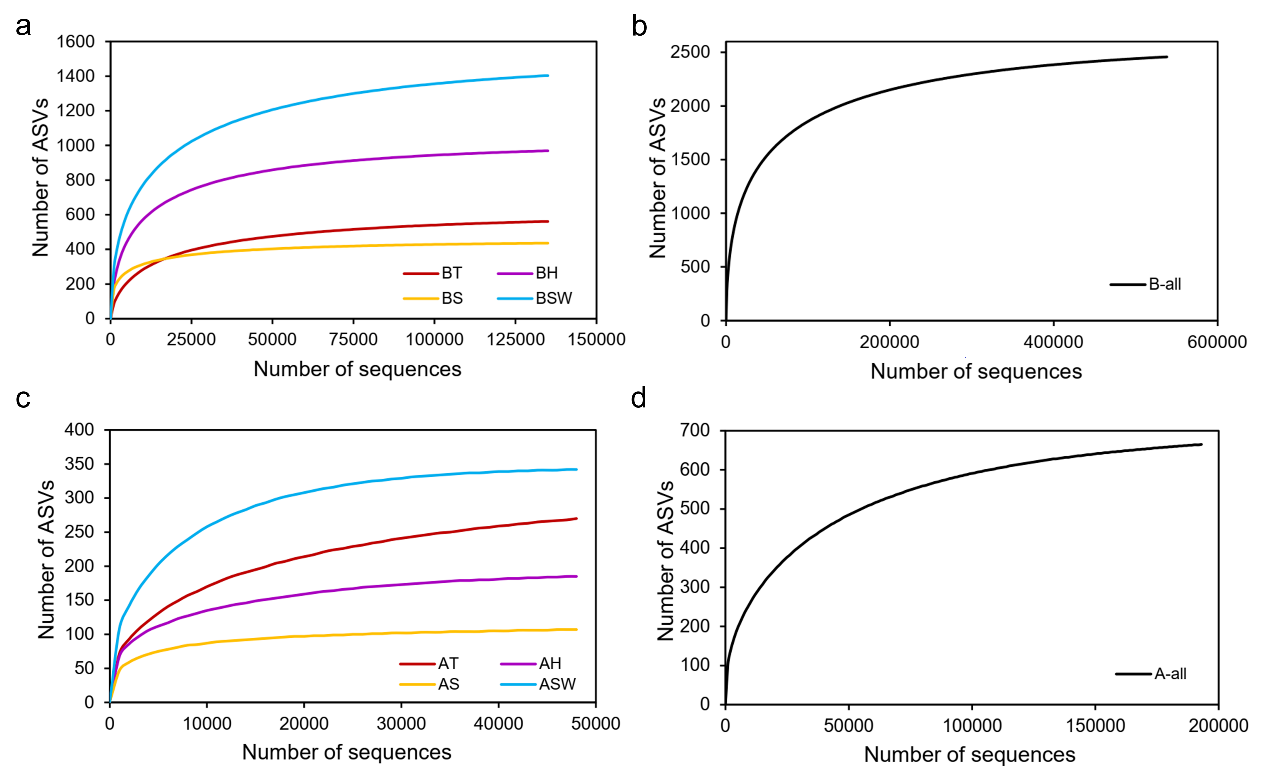


Fig. S2 Rarefaction curves of similarity-based amplicon sequence variants (ASVs). **a, b** bacterial samples and the combined set of 60 samples; **c, d** archaeal samples and the combined set of 60 samples. T=*Tedania* sp., H=*Haliclona simulans*, S=*Spongia officinalis*, SW=seawater, with “B” signifying bacteria and “A” signifying archaea.


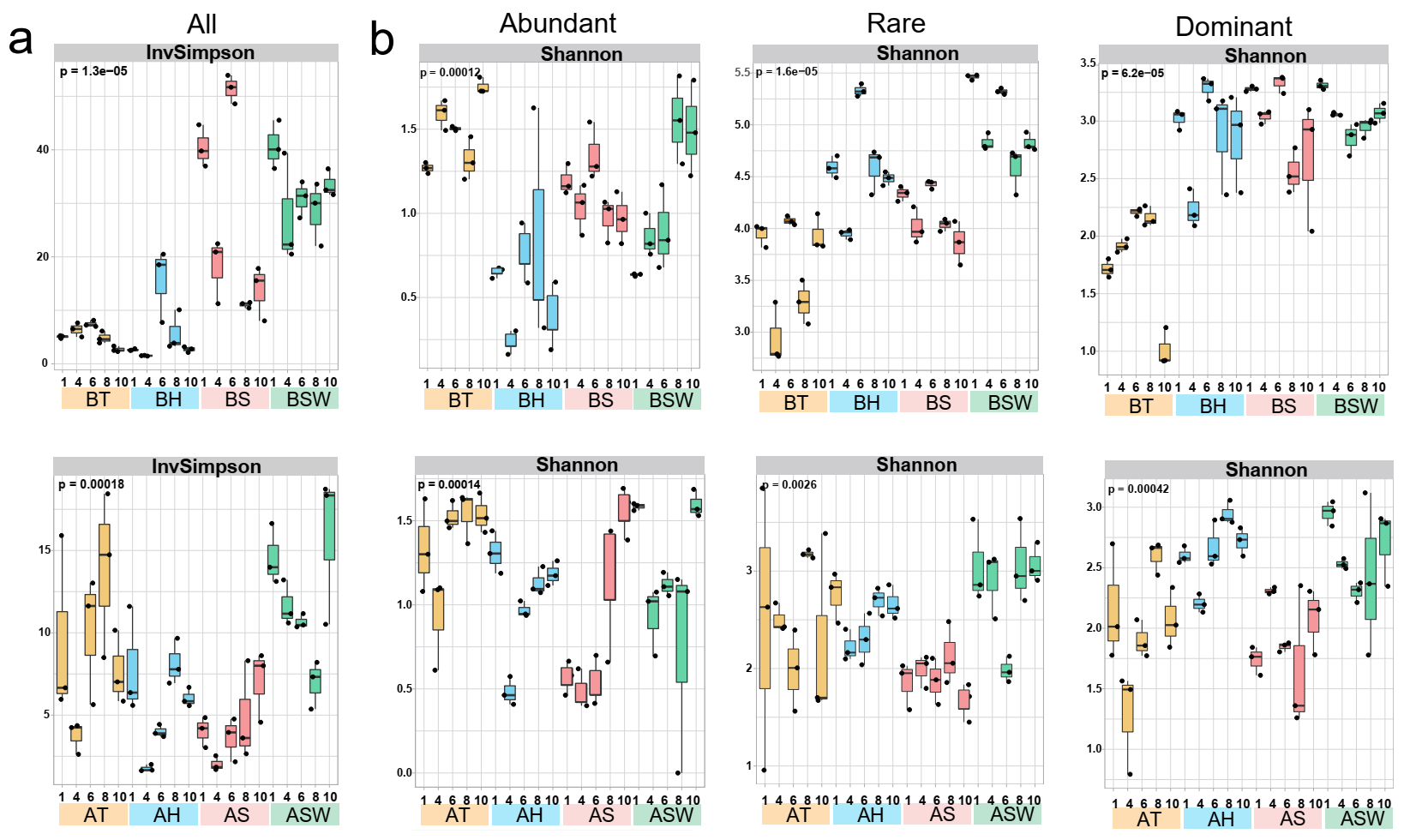


Fig. S3 Alpha diversity of microbial community. **a** Inverse Simpson (InvSimpson) index. **b** alpha diversity of different taxa. T=*Tedania* sp., H=*Haliclona simulans*, S=*Spongia officinalis*, SW=seawater, with “B” signifying bacteria and “A” signifying archaea.


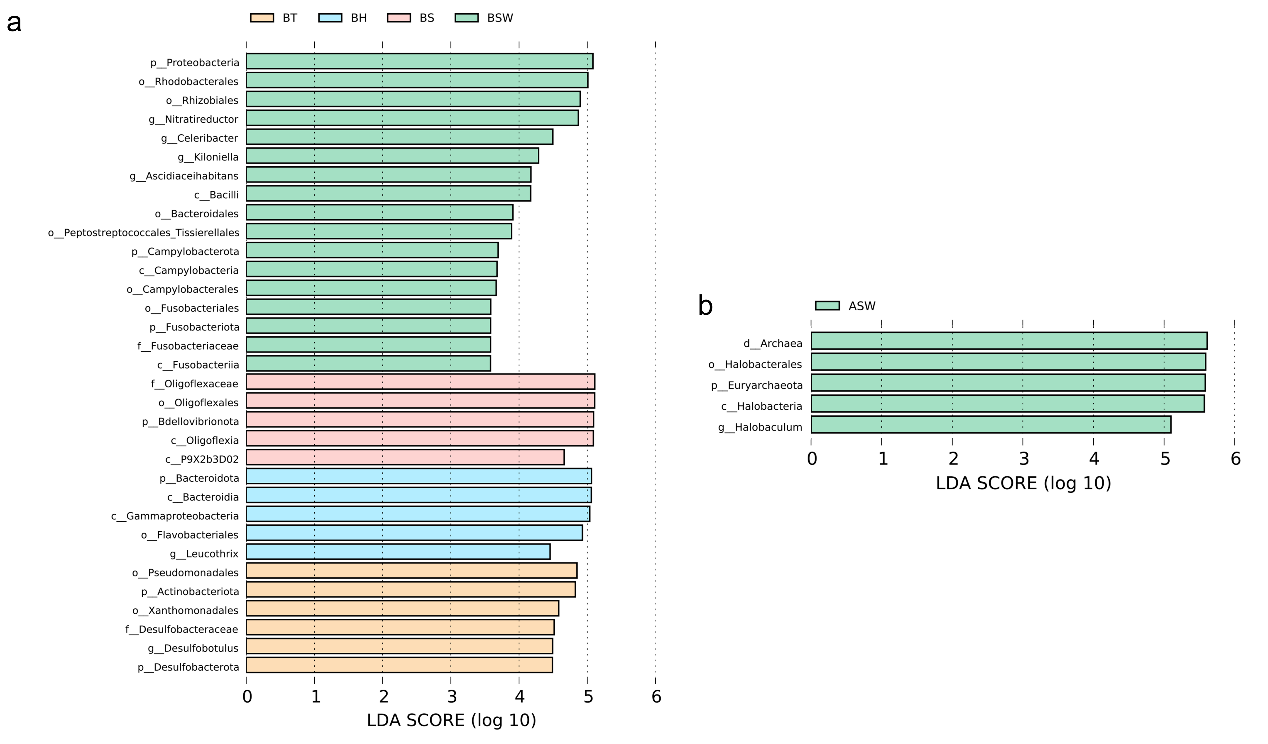


Fig. S4 Linear discriminant analysis effect size (LEfSe) analysis of microbial communities in the sponge and seawater. The figure shows species whose LDA Score is greater than the set value (LDA score > 2, less-strict set to 2; More-strict was set as 4). **a** bacterial community**. b** archaeal community. T=*Tedania* sp., H=*Haliclona simulans*, S=*Spongia officinalis*, SW=seawater, with “B” signifying bacteria and “A” signifying archaea.


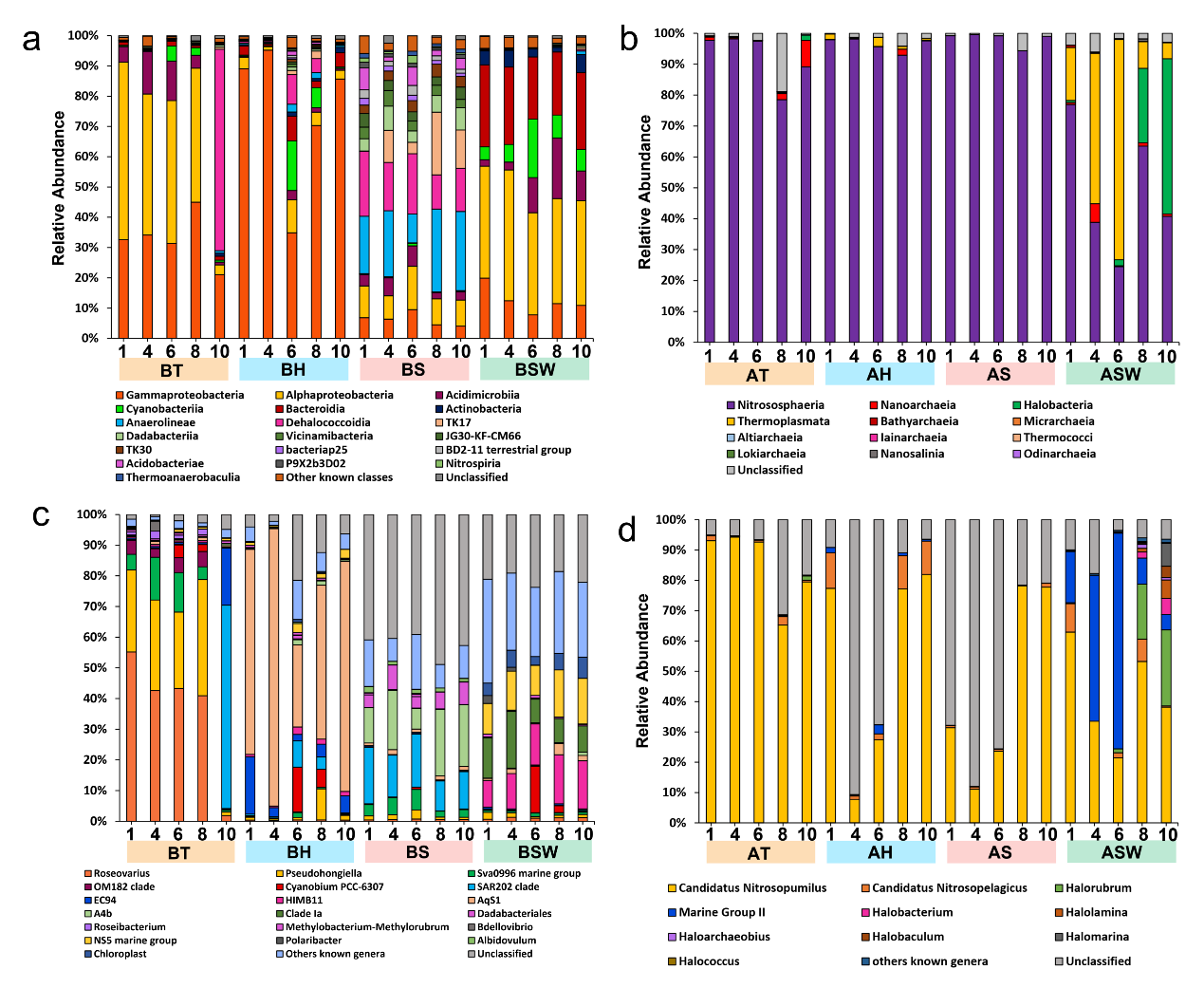


Fig. S5 Microbial community composition under seasonal variation. **a** class level bacteria**. b** genus level bacteria. **c** class level archaea**. d** genus level archaea. T=*Tedania* sp., H=*Haliclona simulans*, S=*Spongia officinalis*, SW=seawater, with “B” signifying bacteria and “A” signifying archaea


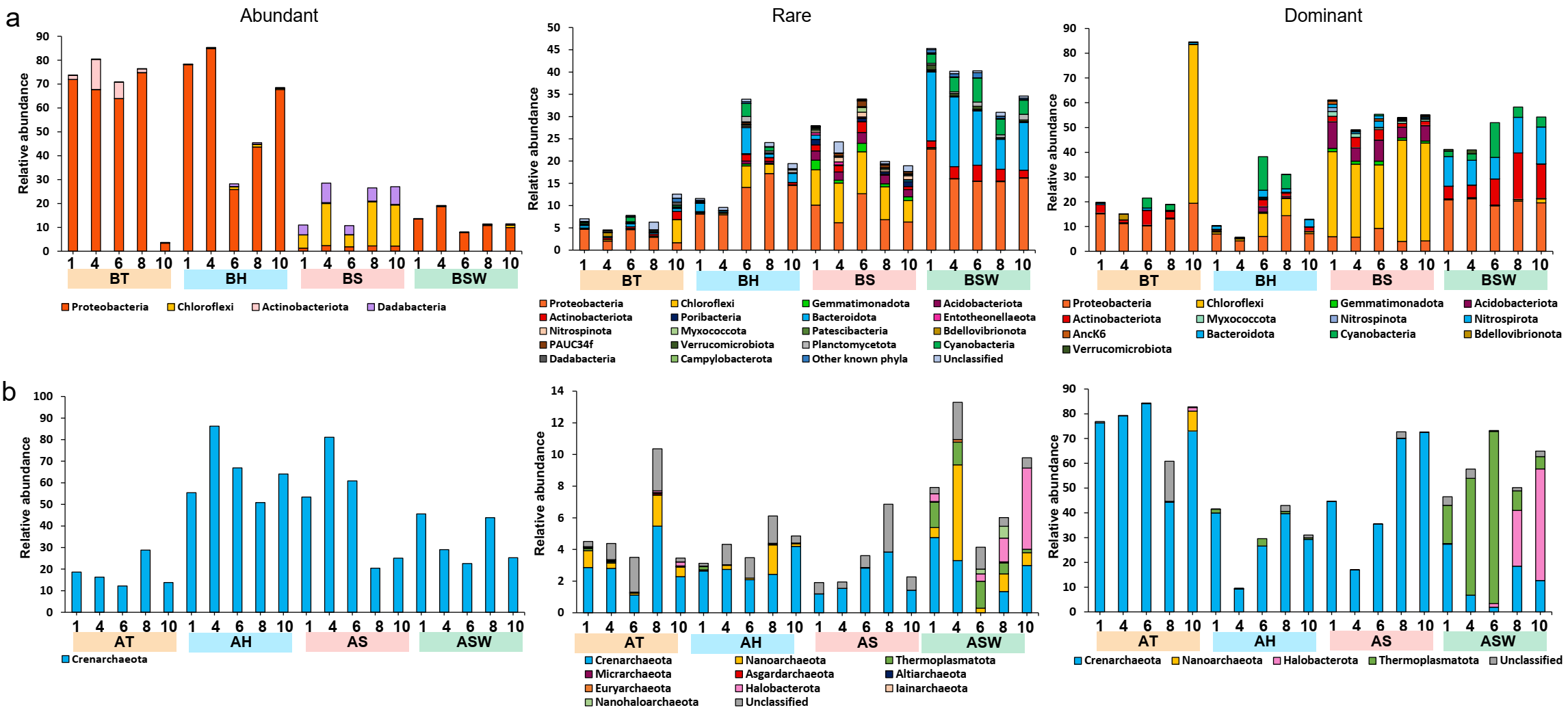
Fig. S6 Different taxa of microbial community composition under seasonal variation. **a** bacterial community**. b** archaeal community. T=*Tedania* sp., H=*Haliclona simulans*, S=*Spongia officinalis*, SW=seawater, with “B” signifying bacteria and “A” signifying archaea.


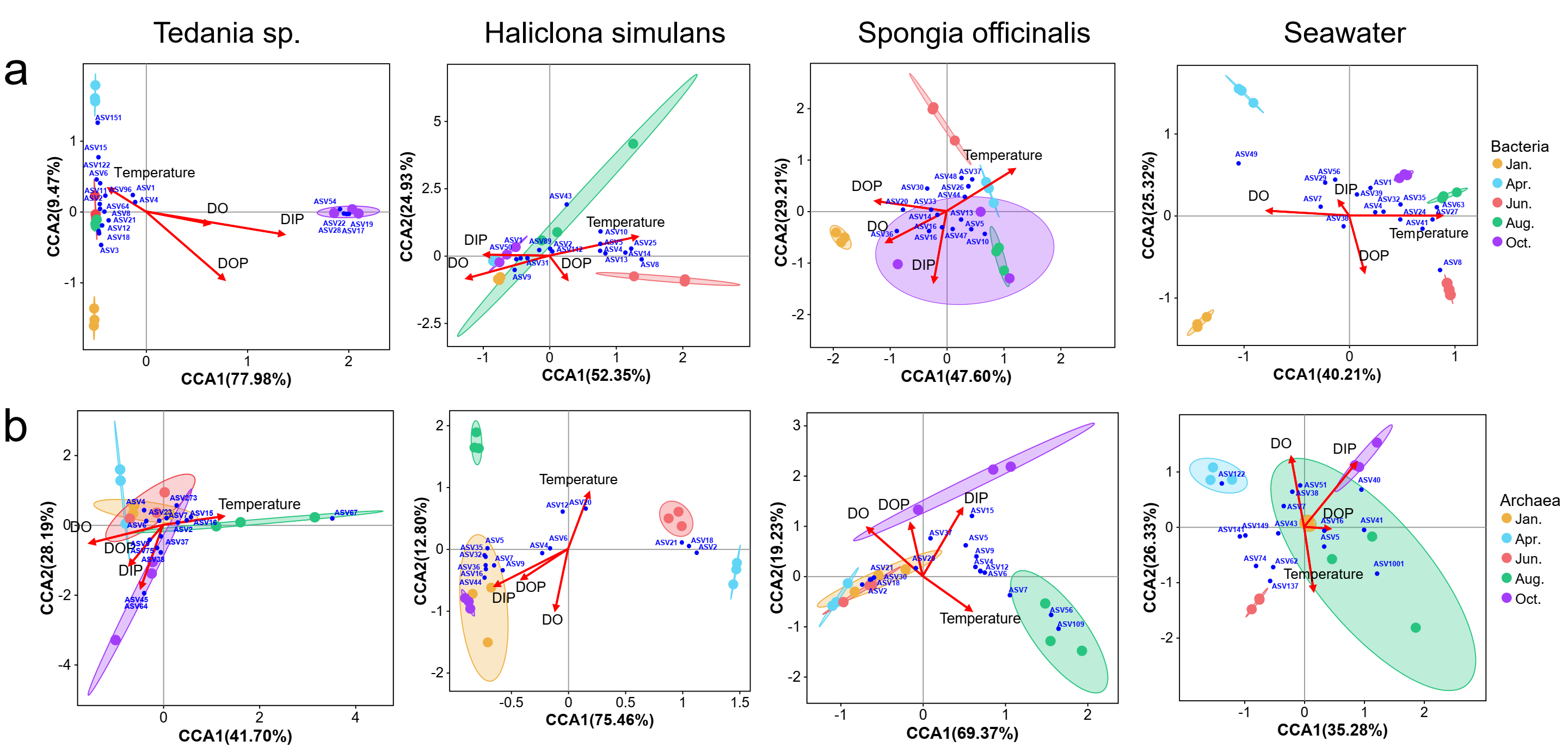


Fig. S7 Microbial community structure and its correlation with environmental factors. **a** bacterial community**. b** archaeal community.


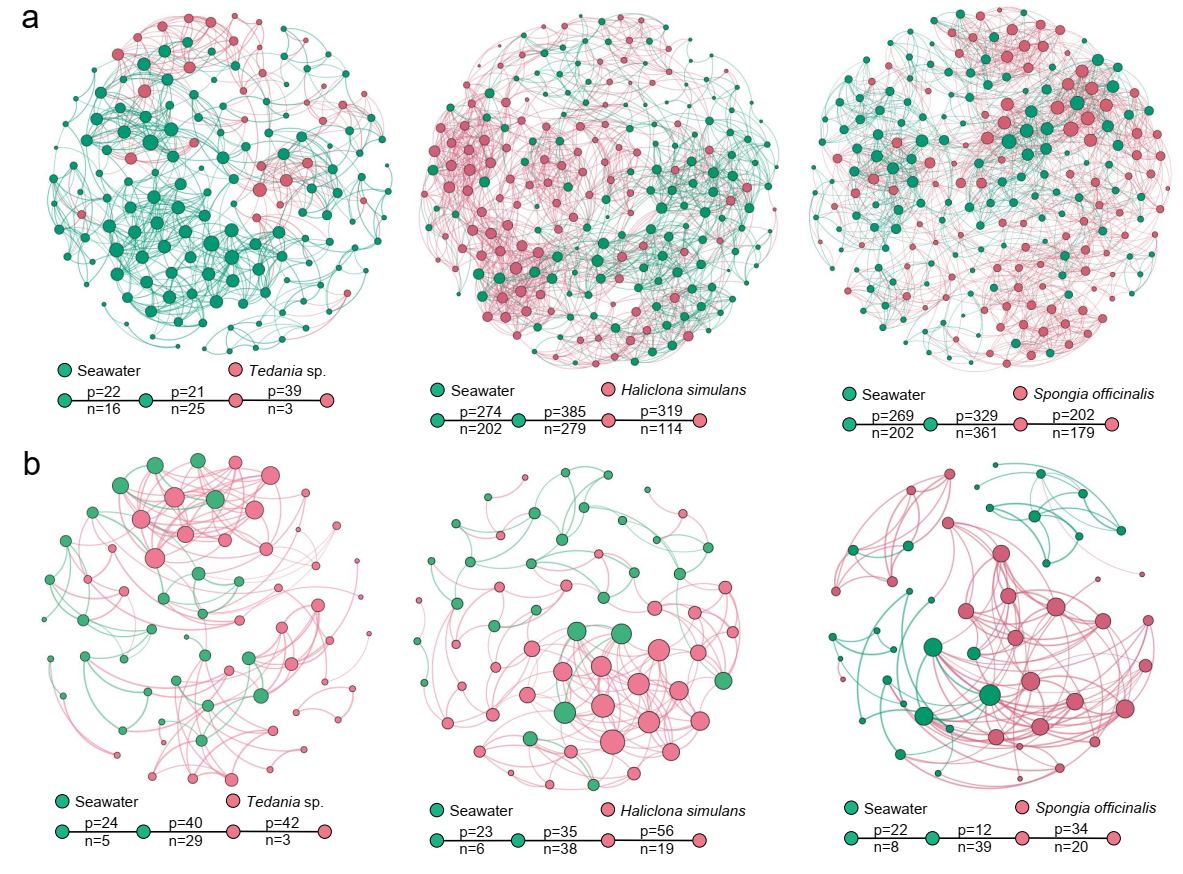


Fig. S8 Interaction between sponge and seawater. **a** bacterial community**. b** archaeal community. The nodes were colored according to different types of taxa. The size of each node is proportional to the number of connections (i.e., degree). p positive edge numbers; n negative edge numbers.


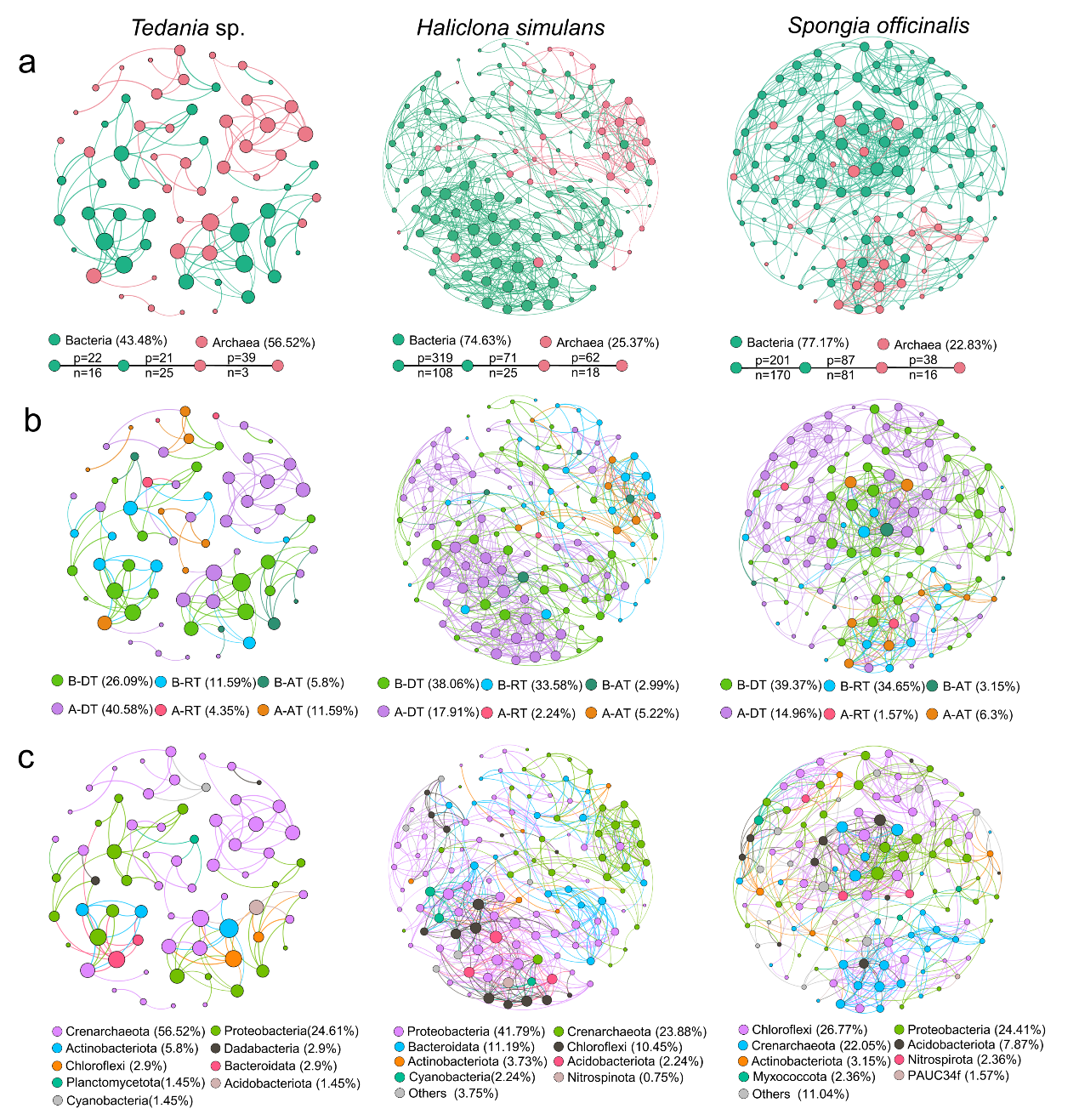


Fig. S9 Bipartite network analysis between bacteria and archaea in the sponges. The nodes were colored according to different types of subcommunity (**a**), taxa (**b**) and community compositions at the phylum level (**c**). The size of each node is proportional to the number of connections (i.e., degree). The numbers in brackets represent the proportion of nodes in different categories. p positive edge numbers; n negative edge numbers. B-DT domianant taxa of bacteria, B-AT abundant taxa of bacteria, B-RT rare taxa of bacteria, A-DT domianant taxa of archaea, A-AT abundant taxa of archaea, A-RT rare taxa of archaea.


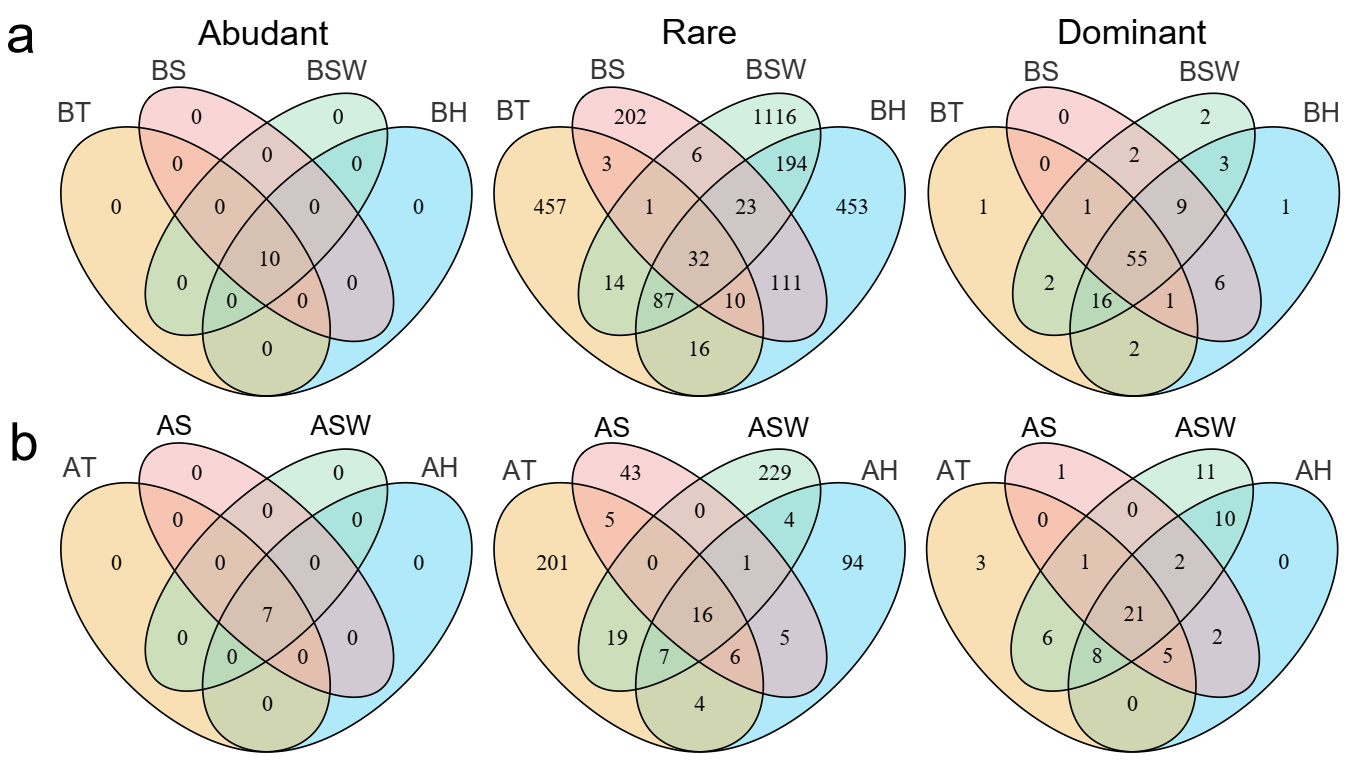


Fig. S10 Venn diagram showing the numbers of unique and shared ASVs of community between sponges and seawater. **a** bacterial community**. b** archaeal community. T=*Tedania* sp., H=*Haliclona simulans*, S=*Spongia officinalis*, SW=seawater, with “B” signifying bacteria and “A” signifying archaea.


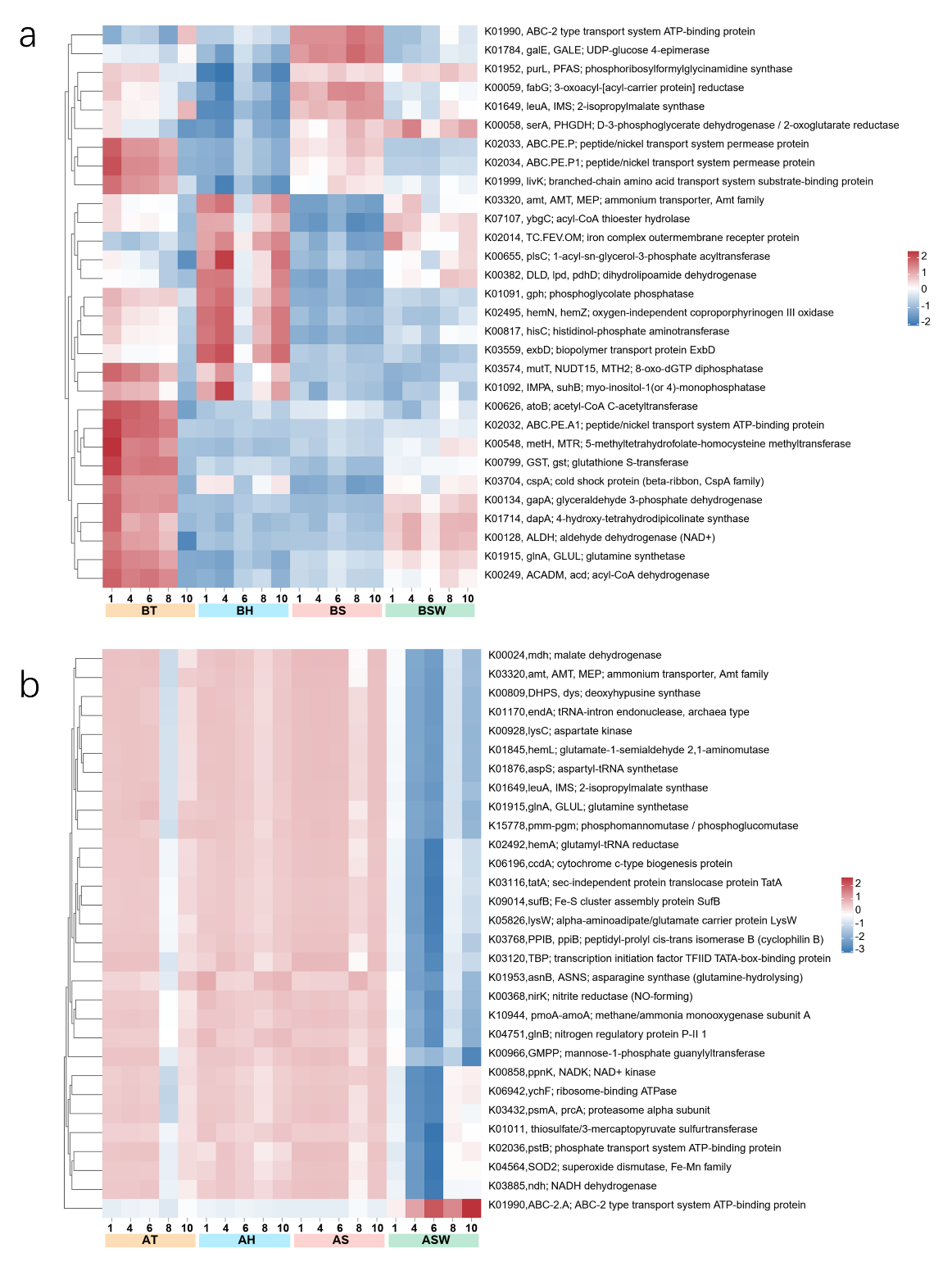


Fig. S11 PICRUSt2 for prediction of metagenome functions about bacteria (**a**) and archaea (**b**) in sponges. T=*Tedania* sp., H=*Haliclona simulans*, S=*Spongia officinalis*, SW=seawater, with “B” signifying bacteria and “A” signifying archaea.


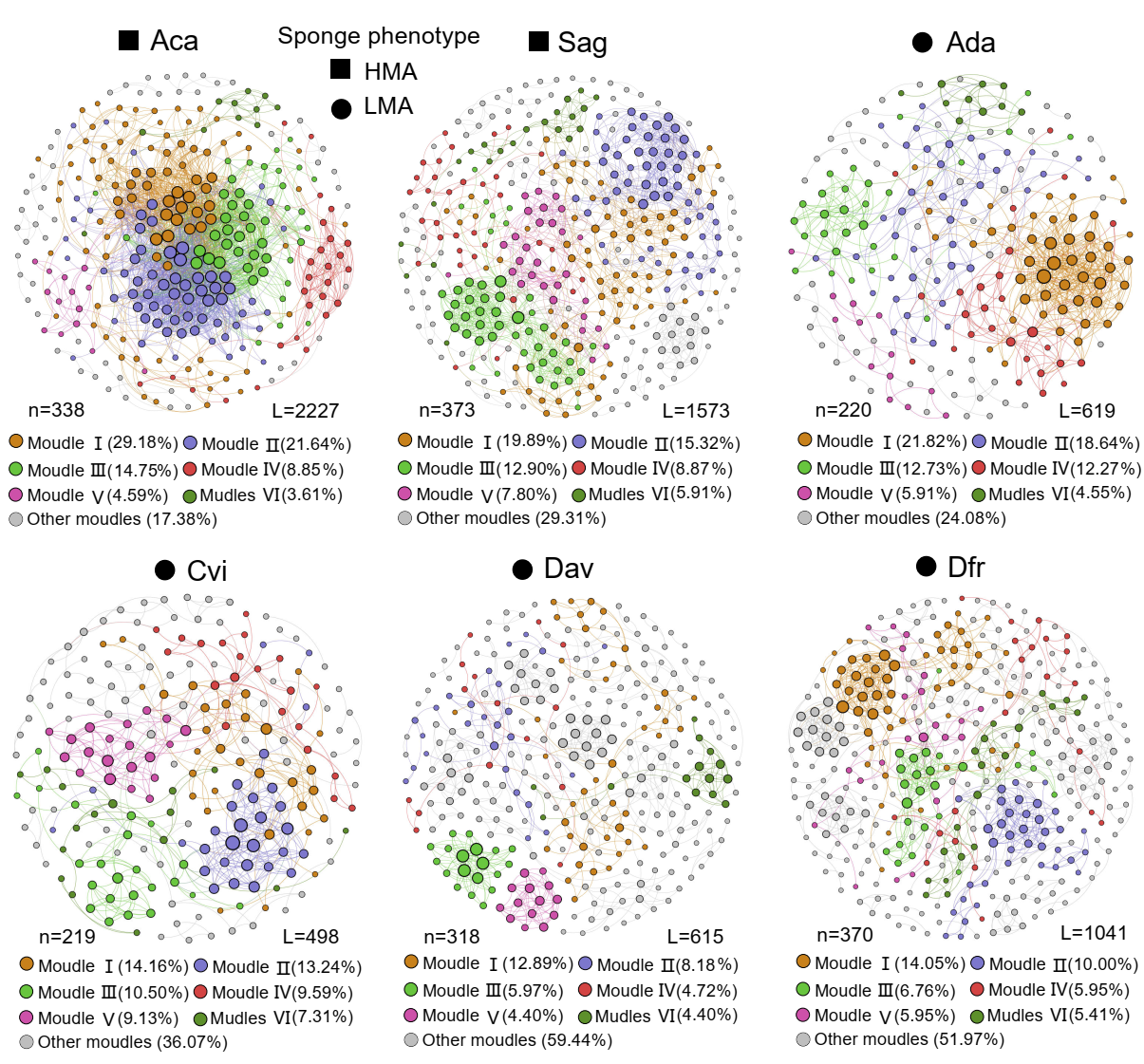


Fig. S12 Interaction of sponge symbionts. The nodes were colored according to different types of modularity classes. The size of each node is proportional to the number of connections (i.e., degree). The numbers in brackets represent the proportion of nodes in different types of modularity classes. n nodes, L links. *Aplysina cavernicola* (Aca), *Spongia agaricina* (Sag), *Axinella damicornis* (Ada), *Cliona viridis* (Cvi), *Dysidea avara* (Dav), *Dysidea fragilis* (Dfr).

**References**

1. Schlitzer R. 2023. Ocean Data View. Available from: http://odv.awi.de
